# Complete genome sequence and comparative genomic analyses of the vancomycin-producing *Amycolatopsis orientalis*

**DOI:** 10.1186/1471-2164-15-363

**Published:** 2014-05-13

**Authors:** Li Xu, He Huang, Wei Wei, Yi Zhong, Biao Tang, Hua Yuan, Li Zhu, Weiyi Huang, Mei Ge, Shen Yang, Huajun Zheng, Weihong Jiang, Daijie Chen, Guo-Ping Zhao, Wei Zhao

**Affiliations:** Nanjing Agricultural University, Nanjing, 210095 China; Shanghai Laiyi Center for Biopharmaceutical R&D, Shanghai, 200240 China; CAS Key Laboratory of Synthetic Biology, Institute of Plant Physiology and Ecology, Shanghai Institutes for Biological Sciences, Chinese Academy of Sciences, Shanghai, 200031 China; State Key Laboratory of Genetic Engineering, Department of Microbiology, School of Life Sciences and Institute of Biomedical Sciences, Fudan University, Shanghai, 200433 China; Shanghai-MOST Key Laboratory of Disease and Health Genomics, Chinese National Human Genome Center at Shanghai, Shanghai, 201203 China; Shanghai Institute of Pharmaceutical Industry, Shanghai, 200040 China; Department of Microbiology and Li Ka Shing Institute of Health Sciences, The Chinese University of Hong Kong, Prince of Wales Hospital, Shatin, New Territories, Hong Kong, SAR China; China HKY Gene Technology Company Ltd, Shenzhen, Guangdong 518057 China; Medical College, Shenzhen University, Shenzhen, Guangdong 518060 China; Computational Biology Center, Memorial Sloan Kettering Cancer Center, New York, NY 10065 USA

**Keywords:** *Amycolatopsis orientalis*, Complete genome sequencing, Molecular taxonomic characteristics, Vancomycin biosynthesis

## Abstract

**Background:**

*Amycolatopsis orientalis* is the type species of the genus and its industrial strain HCCB10007, derived from ATCC 43491, has been used for large-scale production of the vital antibiotic vancomycin. However, to date, neither the complete genomic sequence of this species nor a systemic characterization of the vancomycin biosynthesis cluster (*vcm*) has been reported. With only the whole genome sequence of *Amycolatopsis mediterranei* available, additional complete genomes of other species may facilitate *intra*-generic comparative analysis of the genus.

**Results:**

The complete genome of *A. orientalis* HCCB10007 comprises an 8,948,591-bp circular chromosome and a 33,499-bp dissociated plasmid. In total, 8,121 protein-coding sequences were predicted, and the species-specific genomic features of *A. orientalis* were analyzed in comparison with that of *A. mediterranei.* The common characteristics of *Amycolatopsis* genomes were revealed *via intra*- and *inter*-generic comparative genomic analyses within the domain of actinomycetes, and led directly to the development of sequence-based *Amycolatopsis* molecular chemotaxonomic characteristics (MCCs). The chromosomal core/quasi-core and non-core configurations of the *A. orientalis* and the *A. mediterranei* genome were analyzed reciprocally, with respect to further understanding both the discriminable criteria and the evolutionary implementation. In addition, 26 gene clusters related to secondary metabolism, including the 64-kb *vcm* cluster, were identified in the genome. Employing a customized PCR-targeting-based mutagenesis system along with the biochemical identification of vancomycin variants produced by the mutants, we were able to experimentally characterize a halogenase, a methyltransferase and two glycosyltransferases encoded in the *vcm* cluster. The broad substrate spectra characteristics of these modification enzymes were inferred.

**Conclusions:**

This study not only extended the genetic knowledge of the genus *Amycolatopsis* and the biochemical knowledge of *vcm*-related post-assembly tailoring enzymes, but also developed methodology useful for *in vivo* studies in *A. orientalis*, which has been widely considered as a barrier in this field.

**Electronic supplementary material:**

The online version of this article (doi:10.1186/1471-2164-15-363) contains supplementary material, which is available to authorized users.

## Background

**Figure 1:**
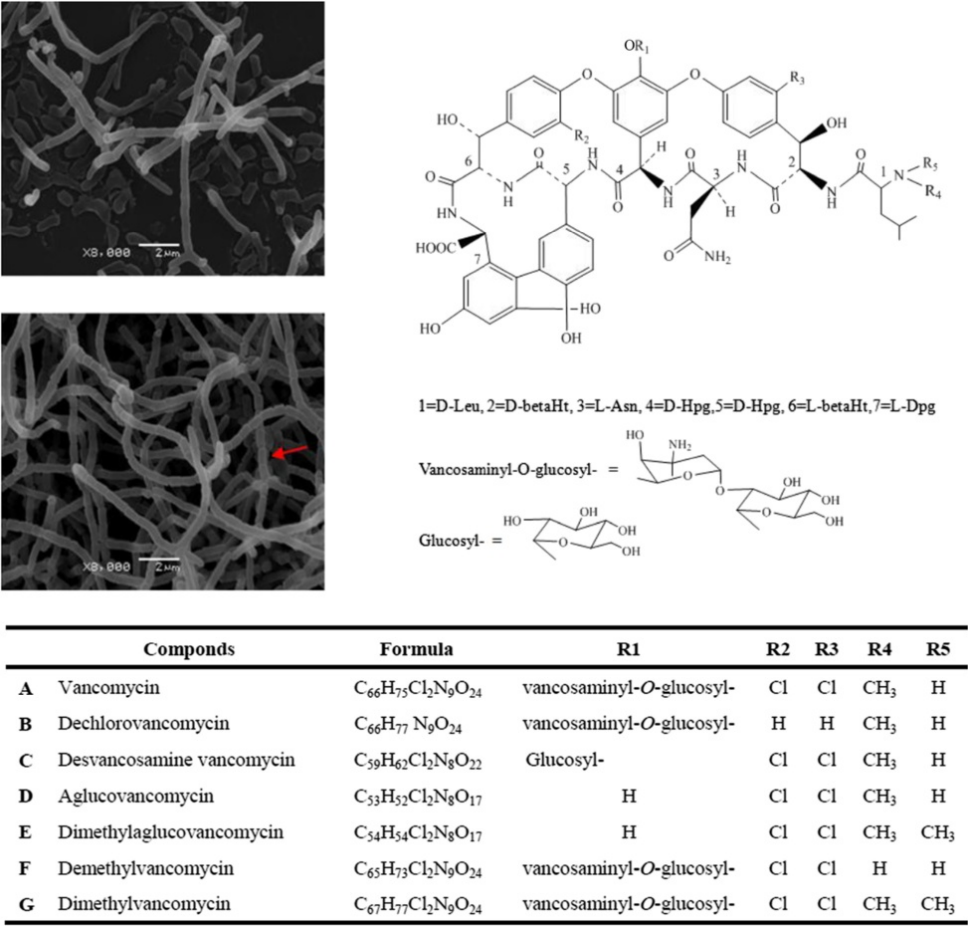
**Morphological differentiation of mycelia in** ***Amycolatopsis orientalis*** **HCCB10007 and chemical structures of vancomycin variants.** Scanning electron micrograph of *A. orientalis* HCCB10007 cultured for one or three incubation days (upper left of the panel). The red arrow indicates the sporulation of *A. orientalis* cultured for three days*.* The core structural formula proposed for vancomycin and its variants (upper right of the panel) shows minor modifications of the heptapeptide core of vancomycin. Table below shows the specific formulae and radical compositions of each vancomycin variant compounds. Alphabetic numbering in the table are corresponding to the legend of Figure 6.

*Amycolatopsis orientalis* is a Gram-positive filamentous actinomycete that produces vancomycin (Figure 1), which is a potent glycopeptide antibiotic that has been used for more than three decades for the treatment of serious methicillin-resistant *Staphylococcus aureus* (MRSA) infections [1]. However, the reports of increased emergence of vancomycin-resistant *S. aureus* (VRSA) and vancomycin-resistant enterococci (VRE) in recent years have presented an urgent challenge to human health, which requires the development of new antibiotics against these pathogens [2–5]. Although some semisynthetic lipoglycopeptide antibiotics, such as telavancin, oritavancin and dalbavancin have been developed recently and their anti-VRSA activities proved *in vitro*[6], the *in vivo* potency of these antibiotics is yet to be demonstrated specifically by clinical studies. Thus, further discovery and development of new glycopeptide type drug candidates continues to be an important mission for biologists and organic chemists.

The gene clusters responsible for the biosynthesis of chloroeremomycin (*cep*) in *A. orientalis* A82846 and balhimycin (*bal*) in *Amycolatopsis balhimycina* DSM 5908, both of which possess an identical heptapeptide backbone and similar antibiotic activities compared with vancomycin, were completely (*cep*, NCBI accession number: AJ223998, AJ223999, and AL078635) or partially (*bal*, NCBI accession number: Y16952) sequenced and annotated about 10 years ago [7, 8]. Thereafter, a series of genes from the two clusters, especially those encoding the post-assembly tailoring enzymes involved in chlorination [9], glycosylation [10–13], and methylation [14], were characterized sequentially. For example, the crystal structures of the TDP-*epi*-vancosaminyltransferase, GtfA [12]; the UDP-glucosyltransferase, GtfB [11]; and the glycopeptide N-methyltransferase, MtfA [14] from *A. orientalis* A82846 were resolved. The methylation function of MtfA from *A. orientalis* A82846 in the synthesis of glycopeptide antibiotics was studied in *Streptomyces toyocaensis*[14], and the halogenase activity of BhaA from *A. balhimycina* DSM 5908 was verified *in vivo*[9]. Of the enzymes encoded by the biosynthetic gene cluster of vancomycin (*vcm*) in *A. orientalis* ATCC 43491, *in vitro* experiments demonstrated that GtfE is responsible for the addition of D-glucose to the hydroxyl of 4-hydroxyphenylglycine and that GtfD can transfer the L-vancosamine moiety to variant glucosyl-peptides as its substrates [10, 15]. In fact, as early as 1997, the *A. orientalis* GtfE was expressed in *S. toyocaensis*, and a hybrid glycopeptide antibiotic, namely glucosyl A47934, was produced [16]. However, unlike *A. balhimycina*, *A. orientalis* is not amenable for genetic manipulations because of difficulties encountered in DNA transformation [17]. Therefore, most genes of the *vcm* cluster have been characterized by heterologous expression or *in vitro* enzymatic/structural analysis [15, 18, 19], with little *in vivo* data reported.

To date, the DNA sequences, along with their annotation information, have been provided for *vcm* cluster genes including those encoding the monooxygenases (NCBI accession number: AF486630.1, FJ532347.1), the halogenase (NCBI accession number: FJ532347.1), the glycosyltransferases (NCBI accession number: U84350.1) and the vancomycin-resistance proteins (NCBI accession number: AF060799.1). Of these, the functions of the monooxygenase (OxyB [19]), glycosyltransferases (GtfE and GtfD [10, 15, 16]), and the vancomycin-resistance proteins (VanHAX [20, 21]) have been well characterized. We cloned and sequenced the whole *vcm* gene cluster in 2010 (NCBI accession number: HQ679900.1). However, with the exception of the glycosyltransferases and their encoding genes [10, 15], other post-assembly tailoring enzymes encoded in the *vcm* cluster, such as the halogenase and the methyltransferase, have been barely experimentally characterized so far and their functions are only assumed based on the similarity of the proteins to those encoded by *bal* or *cep*[9, 14].

The complete genome sequences of the rifamycin producers *Amycolatopsis mediterranei*[22, 23] not only revealed the special genomic features of the genus *Amycolatopsis*, but also confirmed it as a clade of rare actinomycetes potentially rich in antibiotic production capabilities. However, although three draft datasets for the genomes of *A. orientalis* subsp. *orientalis* were released recently [22, 23], neither the annotation nor genomic analysis for these glycopeptide antibiotic-producing *Amycolatopsis* strains is available to date, particularly, at the level of the complete genome sequence. Here, we report the whole genome sequence of an industrial strain (HCCB10007) of *A. orientalis* (CP003410 and CP003411). This strain produces high yields of vancomycin, and is derived from the species type strain ATCC 43491 through series of physical and chemical mutageneses. The high-quality complete genome sequence of *A. orientalis* was compared *intra*- and *inter*-generically to those of its close or distant phylogenetic relatives within the domain of actinomycetes to characterize species-specific and genus-common features of the genomes. Moreover, functions of the predicted halogenase and methyltransferase of the *vcm* cluster in *A. orientalis* were characterized *via* robust spectroscopic analyses in the corresponding site-specific mutants, generated by a customized homologous recombination mutation method.

## Results and discussion

### General and species-specific features of the complete *A. orientalis* genome

**Figure 2:**
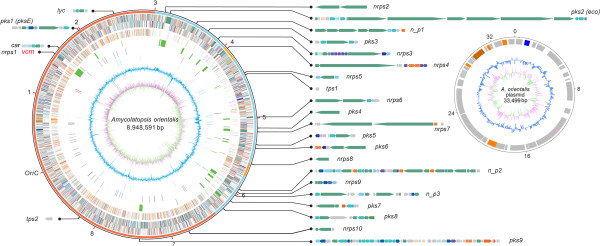
**Genome atlas of the** ***A. orientalis*** **and gene clusters for secondary metabolism.** The large circle represents the chromosome: the outer scale is numbered in megabases and indicates the core (red), quasi-core (orange), and non-core (sky blue) regions. The circles are numbered from the outside in. The genes in circles 1 and 2 (forward and reverse strands, respectively) are color-coded according to COG functional categories. Circle 3 shows selected essential genes (cell division, replication, transcription, translation, and amino-acid metabolism; the paralogs of essential genes in the non-core regions are not included). Circle 4 shows the secondary metabolic clusters, which are further enlarged outside the circle for detailed illustration. The *vcm* cluster is further illustrated in Figure 6. Circle 5 depicts the RNAs (blue, tRNA; red, rRNA). Circle 6 shows the mobile genetic elements (transposase, phage). Circle 7 depicts the GC content. Circle 8 shows the GC bias (pink, values > 0; green, values < 0). The small circle on the right side represents the plasmid DNA sequence. The outer scale is numbered in kilobases. All of the genes, regardless of the forward or reverse strands, are illustrated in the same circle. Circles 2 and 3 are the same as circles 7 and 8 of the large chromosome, respectively.

The genome of *A. orientalis* HCCB10007 comprises two replicons (Figure 2), a large circular chromosome (8,948,591 bp) and a small, dissociated circular plasmid (33,499 bp). The same circular chromosomal topology with that of *A. mediterranei* U32 [24] and *A. mediterranei* S699 [25, 26], which are the other two complete genomes of the *Amycolatopsis* genus currently available, implies that this is a common topological feature that differs from the *Streptomyces* linear chromosomes [27]*.* The genome of *A. orientalis* HCCB10007 is much smaller (1.3 Mbp) than that of *A. mediterranei*, and only 8,121 protein-coding sequences (CDSs) were predicted, which is approximately 1,100 fewer CDSs than those identified in the genome of *A. mediterranei* (Table 1)*.* The difference is mainly accounted for ~1.1 Mbp shorter in the length of the non-core regions of *A. orientalis*. Furthermore, this difference is also enhanced to a certain extent (about 0.2 Mbp) by the smaller average size of the intergenic region (IR) both in the core and the non-core regions of the *A. orientalis* genome (Table 1), resulting in a more compact arrangement of genes (coding density of 90.4%) compared with that of *A. mediterranei* (89.1-89.3%).

**Table 1:** **General features of the** ***Amycolatopsis*** **genomes**

Species		***A. orientalis***		***A. mediterranei***			
Strain		HCCB10007		U32		S699	
**Length (bp)**		8,948,591		10,236,715		10,246,920	
Core/quasi-core vs Noncore (Mbp)		5.9	3.0	6.1	4.1	6.1	4.1
**GC content**		69.0%		71.3%		71.3%	
Core/quasi-core vs Noncore		69.0%	69.0%	71.1%	71.6%	71.1%	71.6%
**ORFs**	Total	8,121		9,228		9,227	
Core/quasi-core vs Noncore		5,624	2,497	5,627	3,601	5,630	3,597
	Proteins with function assigned	5,518 (67.9%)		6,441 (69.8%)		6,386 (69.2%)	
	Hypothetical proteins with unknown function	2,603 (32.1%)		2,787 (30.2%)		2,841 (30.8%)	
	Average ORF size (bp)	996		990		989	
Core/quasi-core vs Noncore		955	1,089	966	1,027	965	1,027
	Average Intergenic region size (bp)	105		119		121	
Core/quasi-core vs Noncore		101	116	115	126	118	126
	Coding density (%)	90.4%		89.3%		89.1%	
Core/quasi-core vs Noncore		90.5%	90.3%	89.4%	89.1%	89.0%	89.1%
**RNA**	rRNA operons	4		4		4	
Core/quasi-core vs Noncore		4	0	4	0	4	0
	tRNA genes	50		52		52	
Core/quasi-core vs Noncore		41	9	41	11	41	11

Initiated from *oriC*, the *dnaA* gene was chosen as the starting point for the numbering of the total CDSs in clockwise order (Figure 2). We assigned 5,518 CDSs (67.9%) to known or putative functions, whereas the remaining 2,603 CDSs (32.1%) were annotated as genes encoding hypothetical proteins (Table 1). The dissociated plasmid (designated as pXL100) encodes 49 genes, of which 43 are functionally unknown. Similar to *A. mediterranei*, the *A. orientalis* genome contains four rRNA operons (16S-23S-5S), and their 16S RNA sequences are at the range of 97% identical (Additional file 1: Table S1). Comparing the four rRNA operons within the *A. orientalis* genome, the first two, counted clockwise from *dnaA*, are both transcribed in the forward direction and their 16S rRNA sequences are slightly different (98–99% identity). The second two are transcribed from the reverse strand and share identical sequences for their 16S rRNAs (Additional file 1: Table S1). *A. orientalis* has 50 tRNA genes, which are largely similar to those of *A. mediterranei*, both in the chromosomal location and anticodon constitutions, with only a few exceptions, such as one less arginine and tyrosine tRNA genes and one more glutamic acid tRNA gene. It is worth emphasizing that, unlike *A. mediterranei*, no selenocysteine tRNA (tRNA^Sec^) was found in the *A. orientalis* genome. Correspondingly, genes encoding selenocysteine synthase (*selA*), elongation factor (*selB*), and selenophosphate synthase (*selD*) were not found in the *A. orientalis* chromosome. Formate dehydrogenase, which has a selenocysteine (Sec)-encoding UGA codon found in the *A. mediterranei* genome, is also absent in *A. orientalis*. Compared with *A. mediterranei*, *A. orientalis* demonstrates a clearer sporulation phenotype (Figure 1). Although the genes responsible for this phenotypic difference are yet to be thoroughly defined, two genes, *spsF* (AORI_0253) and *spsG* (AORI_0254), encoding spore coat proteins, were identified only in the genome of *A. orientalis*. In contrast, the two pMEA100-like integrated plasmids found in the *A. mediterranei* genomes are absent from the genome of *A. orientalis*, whereas the free plasmid pXL100 present in *A. orientalis* HCCB10007 is not found in any other sequenced *Amycolatopsis* strains.

Reciprocal BLASTP was used to calculate the orthologs between *A. orientalis* and other related actinomycetes (*A. mediterranei* S699 and U32, *Amycolatopsis* sp. ATCC 39116, *Saccharopolyspora erythraea*, *Streptomyces coelicolor*, *Saccharomonospora viridis*, *Nocardia farcinica*, and *Mycobacterium tuberculosis*; Additional file 1: Figure S1). By employing a relatively strict condition (identity > 30%, length coverage > 80%), *A. mediterranei* (U32 or S699) shares 50.3% of the total CDSs (4,642 or 4,650) as orthologs with *A. orientalis*, which is the highest among all of the comparisons of the selected actinomycetes. The genome of *Amycolatopsis* sp. ATCC 39116 was recently sequenced by the DOE Joint Genome Institute (JGI) [28], and a high-quality draft with 11 contigs was released in GenBank (accession no. AFWY00000000). We annotated it online using the fully automated service RAST [29] and found that, for the 8,328 predicted CDSs, *Amycolatopsis* sp. ATCC 39116 shares 4,165 orthologs (50.0%) with *A. orientalis* (Additional file 1: Figure S1) *S. erythraea* shared 2,871 orthologs (39.9%) with *A. orientalis*, which is the second highest number among sequenced actinomycetes other than that of *Amycolatopsis*, coincides with the close phylogenetic relationship between the two genera (*Saccharopolyspora vs. Amycolatopsis*). Although *S. viridis* shares only 2,318 orthologs with *A. orientalis*, with its small chromosome (4.3 Mbp) encoding 3,828 proteins, the genus *Saccharomonospora* represented by *S. viridis* is still considered phylogenetically close to *Amycolatopsis*, sharing an extremely high percentage (approximately 60.6%) of orthologs, even higher than that between any two species of the same genus (Additional file 1: Figure S1).

### Genome configuration and plasticity of *A. orientalis* compared with *A. mediterranei*

The unique chromosomal configuration consisting of core versus non-core regions characterized by the distinct features in gathering of essential genes (*i.e.*, genes coding for functions of cell division, replication, transcription, translation, and amino-acid metabolism) in the corresponding genomic regions was first recognized in the linear genome of *S. coelicolor*[30] and then in the circular chromosome of *S. erythraea*[31]. Recently, a novel “quasi-core” region, with typical core characteristics, was defined within the non-core region of the *A. mediterranei* U32 genome, along with the proposition of three discriminable criteria, including the gathering of essential genes, the discrepancy in coding density of orthologous genes and the co-linearity of the orthologs’ order [24]. In this study, taking the advantage of the availability of the complete genome sequences of two species (*A. orientalis* and *A. mediterranei*) from the same genus, the chromosomal configuration of these species was analyzed using more rigorous statistical methods, and special genomic plasticity related to major antibiotics production was revealed as probable chromosomal recombination events.

**Figure 3:**
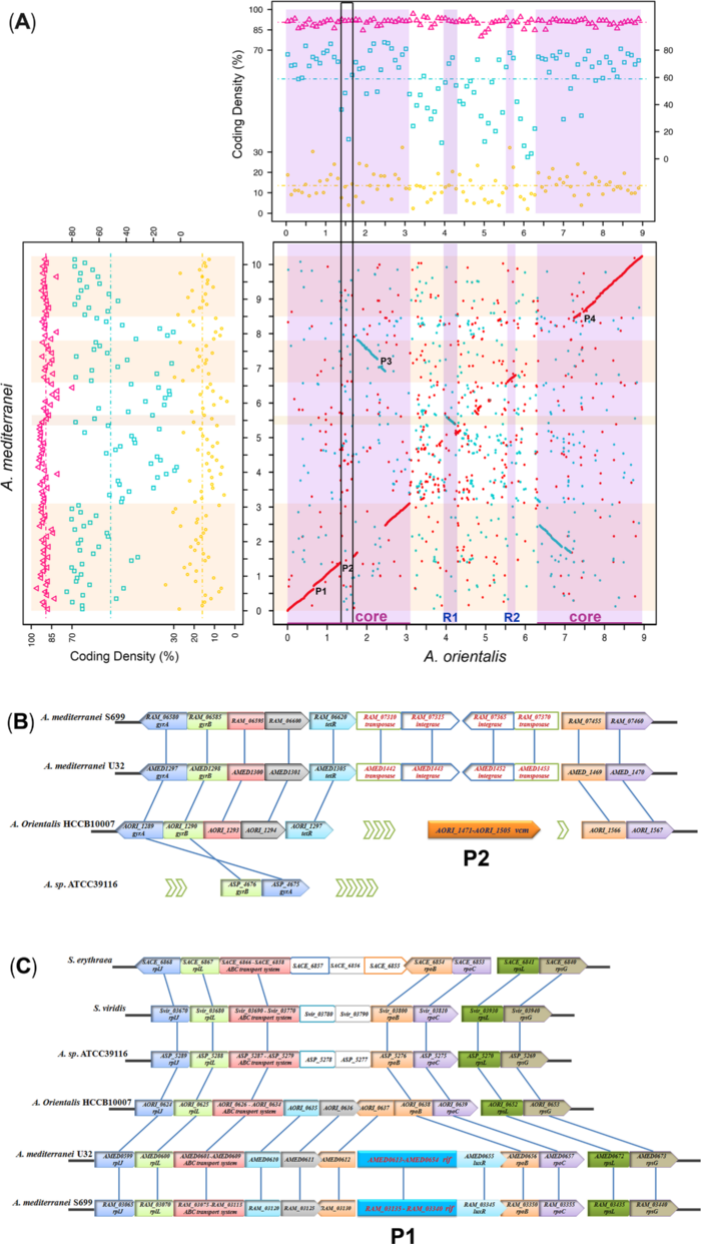
**Genome configurations of** ***A. orientalis*** **and** ***A. mediterranei*** **. (A)** All of the dots in the panels were calculated in a 90-kb sliding window. For the broken X plot (lower right of the panel), the dots represent a reciprocal best match between the genomes of *A. orientalis* and *A. mediterranei*, based on the BLASTP comparison. The X-axis (Y-axis) is the nucleotide scale of the *A. orientalis* (*A. mediterranei*) chromosome. R1 (4.02-4.28 Mb, AORI_3663-AORI_3909) and R2 (5.55-5.75 Mbp, AORI_4997-AORI_5173) were designated as the two quasi-core regions in the *A. orientalis* genome. Reciprocally, two regions (AMED_4864-AMED_5049 and AMED_5970-AMED_7071) were defined as the quasi-core in the *A. mediterranei* genome. The core and quasi-core regions are highlighted in lavender (*A. orientalis*) or in pink (*A. mediterranei*). P1 to P4 were designated as the regions containing biosynthesis clusters of rifamycin (*rif* in *A. mediterranei*), vancomycin (*vcm* in *A. orientalis*), NRPS (*nrps10* in *A. mediterranei*) and polyketide (*pks9* in *A. orientalis*), respectively. In the upper right and lower left panels, the pink triangles represent the coding density of all of the genes; the turquoise squares represent the coding density of orthologs between the genomes of *A. orientalis* and *A. mediterranei*; and the yellow circles represent the coding density of the essential genes. The area within the black square frame is the P2 region containing the *vcm* cluster, with a lower coding density of orthologs and essential genes. **(B)** Alignment of the P2 region with its flanking genes related to the vancomycin biosynthesis in selected actinomycete genomes. The green arrows represent the omitted genes in the corresponding genomes. **(C)** Alignment of the P1 region with its flanking genes related to the rifamycin biosynthesis in selected actinomycete genomes. All of the genome data are available at NCBI.

First, a core region of *A. orientalis* genome (nucleotide coordinates of 0-3.1 Mbp and 6.3-8.9 Mbp, corresponding to AORI_0001-AORI_2890 and AORI_5565-AORI_8121) was recognized by its good co-linearity of the order of its orthologs, with 14.5% of coding density for essential genes compared with 10.2% in non-core regions (*P* < 0.01). Meanwhile, the coding density of orthologous genes in the core region (68.2%) was also higher than that in the non-core regions (37.3%) (*P* < 0.01) (Figure 3A, Additional file 1: Table S2).

Second, different from the analysis between the genomes of *A. mediterranei* and *S. erythraea*, two quasi-core regions (R1 and R2) within the non-core of the *A. orientalis* genomes are defined compared with that of *A. mediterranei* (Figure 3A). The gene orders in these two regions show good conservation with those of *A. mediterranei* and the coding density of orthologous genes is 67.0% (R1, *P* < 0.05) and 73.3% (R2, *P* < 0.01), respectively, significantly higher than that of the non-core regions (Additional file 1: Table S2). In addition, the coding density of essential genes in these two regions is also higher than that in the non-core regions (10.2%), reaching 16.0% (R1, *P* ≈ 0.05) and 22.3% (R2, *P* < 0.05), respectively (employing 45-kb instead of 90-kb sliding window size for statistical analysis, as shown in Additional file 1: Table S2). It is worth mentioning that the identification of two quasi-cores is reciprocal, *i.e*., two regions (AMED_4864-AMED_5049 and AMED_5970-AMED_7071) can be defined as the quasi-cores in the *A. mediterranei* genome by comparison with the genome of *A. orientalis* (Figure 3A), which was obviously unrecognized previously when the genomes of differently related species were compared [24]. In particular, we noticed that all of the four rRNA operons of both species are located in either the core or quasi-core regions (Additional file 1: Table S1 and Figure 2), as are 41 of the 50 tRNA genes of *A. orientalis* (52 of *A. mediterranei*) containing the codons for all 20 essential amino acids (Table 1).

Comparing the genome of *A. orientalis* with that of *A. mediterranei*, a large inversion usually known as the “X pattern” was revealed. Although the order of orthologs is well conserved between these two species, the line of the “X pattern” is not consecutive and is often interspersed with break points. Most of the break points are within the non-core regions encoding the majority of the secondary metabolite biosynthesis gene clusters (Figure 2 and Figure 3A), which might represent some horizontal gene transfer events. The rare break points embedded in the core regions, termed P1 to P4, are usually the regions containing gene clusters for the synthesis of the “species-specific” secondary metabolites, *i.e*., rifamycin (*rif*) in P1 of *A. mediterranei* and vancomycin (*vcm*) in P2 of *A. orientalis* (Figure 3A). The P2 region in *A. orientalis* is nearly 300 kb in length. It not only contains the 64 kb *vcm* cluster, but also encodes many hypothetical proteins or predicted transcriptional regulators, and thus shows a relatively low coding density of orthologs and essential genes. In contrast, the corresponding region of *A. mediterranei* contains dozens of CDSs (over less than 100 kb), including two gene pairs of transposase/integrase (AMED_1442-AMED_1443 and AMED_1452-AMED_1453; Figure 3B). The AMED_1452-AMED_1453 gene pair is a duplicate of AMED_1442-AMED_1443 with a reversed transcription direction, which indicates that an insertion might have occurred in the P2 region of an ancestral strain, which resulted in the acquisition of the *vcm* gene cluster in *A. orientalis*. Unlike the P2 flanking regions, the two regions flanking the *rif* cluster of P1 are highly conserved among the *Amycolatopsis* species (Figure 3C). As indicated by the alignments, the *rif* cluster appears to be inserted between two genes encoding a conserved hypothetical protein (AMED_0612) and the unique DNA-directed RNA polymerase β subunit (RpoB, AMED_0656). Therefore, we speculate that the ancestor of *A. mediterranei* may have acquired the *rif* cluster more recently than that occurred in P2. In addition, a LuxR-like transcriptional regulator (AMED_0655) is located between the 3’ end of the *rif* cluster and the conserved *rpoB* gene (Figure 3C), which seems to have been acquired simultaneously with the *rif* cluster by the ancestral strain. A potential regulatory function of this LuxR-like protein in rifamycin biosynthesis is inferred, and the corresponding experimental proof is currently being pursued (unpublished data).

In this study, *intra*-generic comparative genomic analysis of *Amycolatopsis* not only confirmed the core/quasi-core and non-core genomic configuration, but also discovered certain genomic plasticity hot spots in this genus. It should be noted that the definition of core, quasi-core, or non-core regions of a genome so far remains a relative concept with respect to the genomes of certain species or genera to be compared. The choice of the sliding window size could also influence the characterization of the genomic configuration, which was clearly demonstrated in the case of comparing the coding density of essential genes between the quasi-core and non-core regions. When the window size used in the analysis was reduced from 90-kb to 45-kb, thus doubling the sample size, the *P*-values were reduced from more than one to ≤ 0.05 (Additional file 1: Table S2). In our opinion, these flexible categorizations are somewhat artificial; however, they are useful tools to infer different processes of evolution of a genus or of microevolution of a species. The quasi-core region(s) may represent the residue(s) of the complex evolution dynamics (vertical genomic recombination events) of the ancestral genome, while the non-core regions may represent the chromosomal expansions (horizontal gene transfer) in the various descendants’ genomes. As more whole genome sequences of different strains of one species or different species of one genus, as well as those from closely related genera, are published, the biological implications of this genomic plasticity in bacterial phylogeny will be clarified.

### Development of molecular chemotaxonomic characteristics (MCCs) for the genus of Amycolatopsis

The taxonomic status of *A. orientalis* underwent the same revision history as that of *A. mediterranei*[24]; *i.e.,* it was originally considered a streptomycete [32], then transferred to *Nocardia*[33], and finally classified as a species of the newly established genus *Amycolatopsis*[34], which was typically defined by the biochemical characteristics of its cell wall (chemotype IV) and cell membrane (chemotype II). As initiated in the study of the *A. mediterranei* U32 genome [24], in addition to the molecular genetic basis responsible for the components of arabinose, glycine, diaminopimelic acids and mycolic acids (Additional file 1: Table S3, Additional file 1: Table S4, Additional file 1: Figure S2, and Additional file 1: Figure S3), we attempted to analyze the previously unidentified genetic basis of two more chemotaxonomic phenotypes, *i.e.*, phospholipids and menaquinones.

**Figure 4:**
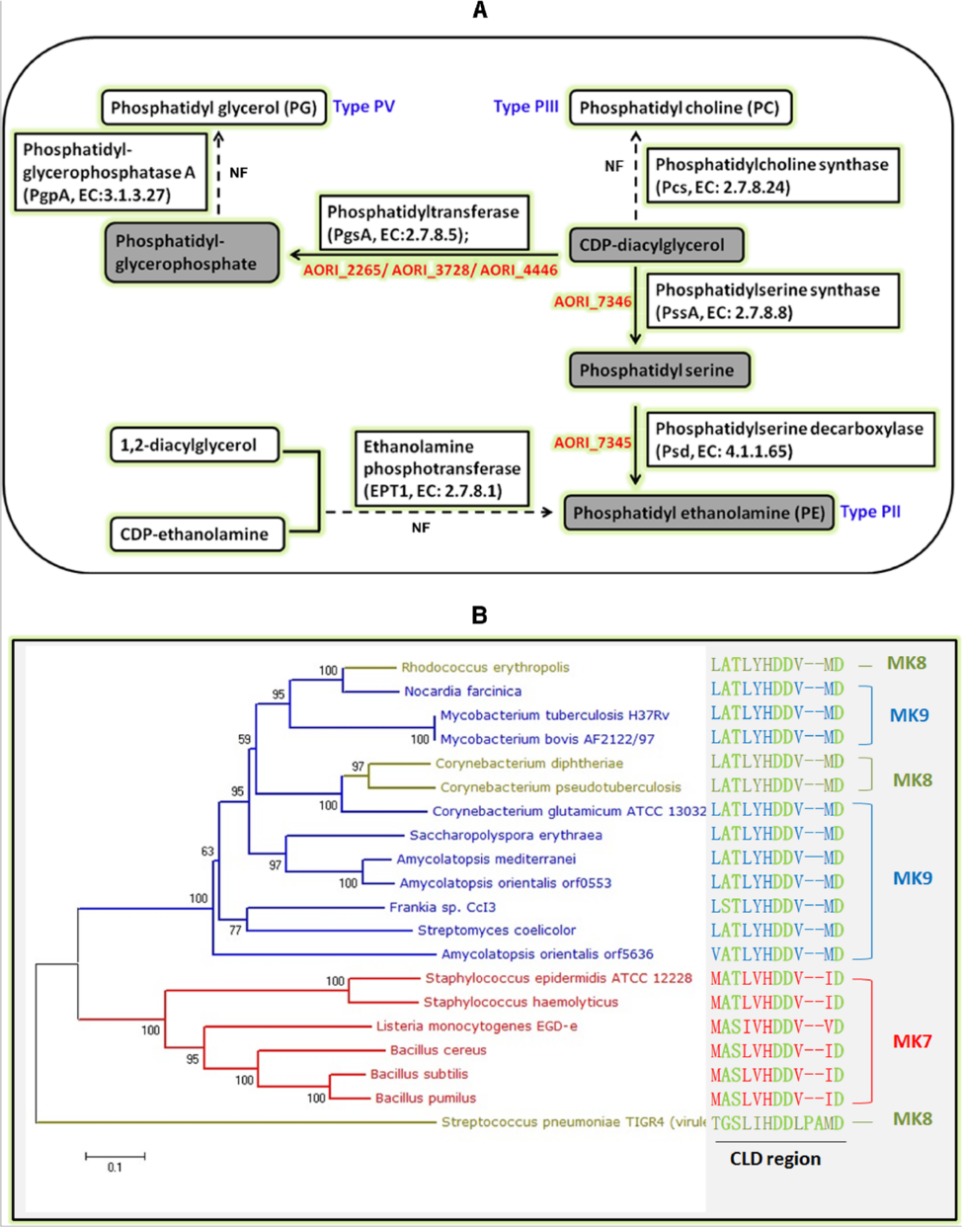
**Biosynthetic pathways of different types of nitrogenous phospholipids in actinomycetes. (A)** The cell membrane of *Amycolatopsis* belongs to the type PII because PE is the dominant phospholipid detected. Two essential proteins (AORI_7345 and AORI_7346, labeled in red color) involved in the biosynthesis of PE were encoded by the *A. orientalis* genome, whereas the genes encoding enzymes involved in other types of nitrogenous phospholipids were not found (NF). Actinomycetes of type PI contain no nitrogenous phospholipids in their cell membrane, while type PII, type PIII, type PIV, and type PV actinomycetes contain the following characteristic phospholipids: PE, PC, GluNU, and PG, respectively. Panel **(B)** illustrates the analysis of isoprenyl diphosphate synthases from type strains of actinomycetes. The names and amino-acid sequences of the strains with different colors represent actinomycetes harboring different-length MKs: red, MK7 (C35); olive-green, MK8 (C40); blue, MK9 (C45). The amino-acid sequences of the chain-length determination (CLD) region are emphasized in green on the right of the panel. The protein sequences were obtained from NCBI at http://www.ncbi.nlm.nih.gov/protein/.

The cell membrane of actinomycetes is classified into five types according to the presence of certain nitrogenous phospholipids [35]. The *Amycolatopsis* cell membrane belongs to the PII type because only one nitrogenous phospholipid, namely phosphatidyl ethanolamine (PE), was usually detected in its cell membrane [35]. In prokaryotes, phosphatidylserine is first generated from CDP-diacylglycerol, a general intermediate for the synthesis of different types of phospholipids, catalyzed by phosphatidylserine synthase (PssA, EC: 2.7.8.8), and is then transformed into PE [36, 37] by phosphatidylserine decarboxylase (Psd, EC: 4.1.1.65) (Figure 4A). Orthologs of both *pssA* (AORI_7346) and *psd* (AORI_7345) could be identified in the *A. orientalis* genome. These two genes also exist in other actinomycetes with a type PII cell membrane, such as *A. mediterranei*, *N. farcinica*, *S. coelicolor*, and *M. smegmatis*, but are absent in actinomycetes with a type PI membrane (no nitrogenous phospholipids) or other types of cell membranes. Moreover, in the genomes of neither *A. orientalis* nor *A. mediterranei* did we identify genes encoding phosphatidylcholine synthase (Pcs, EC: 2.7.8.24), which catalyzes the formation of phosphatidyl choline (PC), the characteristic type PIII phospholipid, or the genes encoding phosphatidylglycerophosphatase A (PgpA, EC: 3.1.3.27), which catalyzes the formation of phosphatidyl glycerol (PG), the characteristic type PV phospholipid (Figure 4A). It is worth mentioning that the gene *ept1*, which encodes ethanolamine phosphotransferase (EPT1, EC: 2.7.8.1) that catalyzes the biosynthesis of PE from 1, 2-diacylglycerol and CDP-ethanolamine in eukaryotes, is also absent in any of the sequenced actinomycete genomes (Figure 4A).

Isoprenoid quinones comprise a hydrophilic head and an apolar isoprenoid side chain, functioning mainly as electron and proton carriers in photosynthetic and respiratory electron transport systems [38]. These compounds have also been used as conventional biomarkers in bacterial chemotaxonomy since the 1960s [39, 40]. In the synthesis of isoprenoid quinones, isoprenyl diphosphate synthase (Isp) catalyzes the consecutive condensation of isopentenyl diphosphate (IPP) with allylic diphosphates and produces a variety of prenyl diphosphates with different chain lengths [38]. Previous studies reported that the specific amino-acid residues of isoprenyl diphosphate synthases attributable to the chain-length determination were designated the chain-length determination (CLD) region [41]. However, for the biosynthesis of isoprenoid quinones with longer chain lengths (more than C30), the consensus CLD region in isoprenyl diphosphate synthases has yet to be clarified [41]. In actinomycetes, menaquinone (MK) is the characteristic type of isoprenoid quinone in the cell membrane. We compared the amino-acid sequences (particularly the CLD region) of Isp from type strains of actinomycetes harboring different-length MKs but no regular patterns could be found (Figure 4B). Hence, the isoprenyl diphosphate synthases were analyzed phylogenetically using the neighbor joining (NJ) method. As shown in Figure 4B, species with MK-7 (C35) in their membranes are clustered within one clade, whereas species with MK-8 (C40) or MK-9 (C45) are clustered together and are indistinguishable in the tree. In addition, the maximum parsimony (MP) method was also used to construct a phylogenetic tree. However, the clustering result could not distinguish the species harboring MK-8 or MK-9 either (Additional file 1: Figure S4). Therefore, it is yet to be experimentally clarified whether the genotypes of isoprenyl diphosphate synthases, *i.e.*, their sequence specificities, are a sufficient determinant for all different side chain lengths of isoprenoid quinone, or whether the variation of isoprenoid quinones in actinomycetes is a quantitative, rather than a qualitative, property that might be determined by gene expression regulation or other post transcription/translational modifications.

In summary, our sequential studies in two species of *Amycolatopsis* (ref to [24] and this work) indicate that the chemotaxonomic characteristics of this genus, which relate to, but differentiate from *Streptomyces* and *Nocardia*, are intrinsically determined by the molecular phylogeny of their encoding genes. On the other hand, the failure to precisely determine the molecular genetic mechanisms underlying the chain-length of MK hinted at the complexity of these genotype/phenotype correlations. Together with some more important chemotaxonomic characteristics, such as the composition of fatty acids, these complex phenotypes and their related molecular genetic mechanisms may prompt further biochemical and molecular biological studies. Nowadays, we propose that, based on whole genome analysis of multiple bacterial strains belonging to and related with a taxon (particularly, species or genus), potential **m**olecular **c**hemotaxonomic **c**haracteristics (MCCs) could be developed as the genotypes underlie the biochemical characteristics (phenotypes) of the taxon. The implementation of MCCs in bacterial systematics will not only alleviate the tedious workload of chemotaxonomic identification, but also improve our understanding of the genetics of bacterial metabolomes, which will form an indispensable portion of the modern prokaryotic taxonomy in the era of genomics [42].

### Biosynthesis of secondary metabolites and the post-assembly modifications of vancomycin in A. orientalis

Twenty-six secondary metabolite biosynthetic gene clusters were predicted in the complete genome of *A. orientalis* HCCB10007, including nine type I polyketide synthase (PKS) clusters, one type II PKS cluster, ten non-ribosomal peptide synthetase (NRPS) clusters, three hybrid PKS-NRPS clusters, two clusters for terpenoids, one cluster for lycopene (*lyc*), and one cluster for β-carotene (*car*) (Figure 2). The total length of these gene clusters was estimated ~552 kb, which is 6.2% of the whole genome. In contrast to the essential genes, most of the secondary metabolite biosynthetic gene clusters (18 out of 26) were in the non-core regions (Figure 2).

To determine the possible phylogenetic relationships of the secondary metabolites biosynthesis gene clusters, all of the CDSs for PKSs, NRPSs, or terpene synthases were compared against the NCBI database *via* BLASTP. The best hits information is provided in Additional file 1: Table S5. Twenty-seven genes in nine biosynthetic gene clusters (34.6% of the total 26 clusters) have orthologs in the *A. mediterranei* genome with the best hitting scores, *i.e.*, *car*, *pks1*, *lyc*, and *tps2* in the core region and *pks3*, *tps1*, *nrps7*, *pks5*, and *pks6* in the non-core regions. Furthermore, the *nrps7*, *pks5*, and *pks6* gene clusters are closely located in the non-core region, particularly the *pks5* and *pks6* clusters (Figure 2). These close correlations between sequence similarity and genomic loci gathering may indicate a common phylogenetic origin.

Notably, among the eight gene clusters for secondary metabolism located in the core and quasi-core regions, except for four clusters (*car*, *pks1*, *lyc*, and *tps2*) orthologous to those encoded in *A. mediterranei* genome, all of the other *A. orientalis* specific clusters (*vcm*, *pks9*, *nrps10*, *nrps4*) are located in the break point of the chromosomal “X pattern” blocks. However, because of the small coding size of *nrps10* and *nrps4*, only the *vcm* cluster (64 kb), located in the P2 break point, and the *pks9* cluster (AORI*_*6587-6642, 61.7 kb), located in the P4 break point, could be traced in the “X pattern” blocks (Figure 3A). The KS domains of *pks9* are similar to those of the salinosporamide A biosynthetic gene cluster in *Salinispora tropica* CNB-440 (73% identity) [43]. This cluster is rich in genes encoding modification enzymes, such as glycosyltransferases, halogenase, and cytochrome P450, which suggests the production of a glycosidic and halogenic compound.

In the non-core regions, cluster *pks2* (AORI_2937-2956, 79.6 kb) contains a type I polyketide synthase, which was once reported to synthesize a glycosidic polyketide ECO-0501 that shows activities against MRSA and VRE [44]. For the other secondary metabolite biosynthesis gene clusters in *A. orientalis* genome, their putative substrates or probable products were predicted using catalytic domain analysis against the SBSPKS [45] or NRPSDB [46] databases and the results are listed in Additional file 1: Table S5. We isolated the total RNA of *A. orientalis* from two different cultures (fermentation medium F1 and nutrient medium F5, Additional file 1: Supplementary Materials and Methods), and used reverse-transcription PCR to detect the transcription profiles of the gene clusters that might synthesize potential secondary metabolites. As shown in the Additional file 1: Figure S5, in both F1 and F5 media, the genes of three clusters (*pks5, n_p2*, and *vcm*) showed significant levels of transcription, with *vcm* being the highest among all gene clusters tested. Another cluster (*nrps4*) was expressed in the F1 fermentation medium but not in the F5 medium. Although we failed to identify any novel secondary metabolites, our data provides a foundation for further exploration.

The *vcm* cluster was annotated to encode a total of 35 enzymes (AORI_1471 to AORI_1505), including three vancomycin-resistance proteins (VanH, VanA, and VanX [7, 8]), three large NRPSs, several post-assembly tailoring enzymes, and a series of biosynthetic proteins for the supply of amino-acid precursors (Table 2). Different from the *cep* and *bal* clusters, in which three genes encoding glycosyltransferases were predicted [7, 8], only two glycosyltransferases are encoded in the *vcm* cluster (AORI_1486 and AORI_1487). On the other hand, the vancomycin-resistance genes *vanHAX* (AORI_1471-AORI_1473) are only predicted in *vcm* and not in the other two clusters (Table 2). Throughout the *A. orientalis* genome, we identified another *vanA* (AORI_8112) and *vanX* (AORI_2227), as well as a two-component system (AORI_7254-AORI_7255) similar to the *vanSR* of *bal* that may be related to the vancomycin resistance.

**Table 2:** **Annotation of the** ***vcm*** **cluster in** ***A. orientalis*** **and the comparison with** ***bal*** **and** ***cep***

AORI_CDS	Gene name	Annotation	Best hit genes (NCBI accession no.)	Identity %
**AORI_1471**	*vanH*	D-lactate dehydrogenase	**-**	**-**
**AORI_1472**	*vanA*	D-alanine-D-alanine ligase	**-**	**-**
**AORI_1473**	*vanX*	D-alanyl-D-alanine dipeptidase	**-**	**-**
**AORI_1474**		Hypothetical protein	**-**	**-**
**AORI_1475**	*vtr*	Regulator protein	lcl|Y16952.3_cdsid_CAG25754.1	83.33
**AORI_1476**	*pdh*	Prephenate dehydrogenase	lcl|Y16952.3_cdsid_CAG25755.1	84.12
**AORI_1477**		ATP-binding cassette, subfamily B	lcl|AJ223999.1_cdsid_CAA11793.1	88.89
**AORI_1478**	*vcmA*	Non-ribosomal peptide synthetase	lcl|AL078635.1_cdsid_CAB45052.1	81.31
**AORI_1479**	*vcmB*	Non-ribosomal peptide synthetase	lcl|AJ223999.1_cdsid_CAA11795.1	82.34
**AORI_1480**	*vcmC*	Non-ribosomal peptide synthetase	lcl|Y16952.3_cdsid_CAC48362.1	86.81
**AORI_1481**	*mbtH*	MbtH protein	lcl|Y16952.3_cdsid_CAC48363.1	89.86
**AORI_1482**	*oxyA*	Cytochrome P450	lcl|Y16952.3_cdsid_CAA76547.1	86.19
**AORI_1483**	*oxyB*	Cytochrome P450	lcl|Y16952.3_cdsid_CAA76548.1	87.44
**AORI_1484**	*oxyC*	Cytochrome P450	lcl|AJ223998.1_cdsid_CAA11791.1	91.69
**AORI_1485**	*vhal*	Halogenase	lcl|Y16952.3_cdsid_CAA76550.1	93.89
**AORI_1486**	*gtfD*	Vancosaminyl transferase	lcl|Y16952.3_cdsid_CAA76553.1	69.93
**AORI_1487**	*gtfE*	Glycosyl transferase	lcl|Y16952.3_cdsid_CAA76552.1	81.17
**AORI_1488**	*vcaC*	Methyltransferase family protein	lcl|Y16952.3_cdsid_CAC48364.1	93.87
**AORI_1489**		LmbE family protein	lcl|Y16952.3_cdsid_CAC48365.1	75.91
**AORI_1490**	*vmt*	Methyltransferase	lcl|AJ223998.1_cdsid_CAA11779.1	73.21
**AORI_1491**	*hpgT*	4-hydroxyphenylglycine aminotransferase	lcl|AJ223998.1_cdsid_CAA11790.1	89.7
**AORI_1492**	*vhp*	Hydrolase	lcl|AJ223998.1_cdsid_CAA11784.1	85.87
**AORI_1493**	*vcmD*	Non-ribosomal peptide synthetase	lcl|AJ223998.1_cdsid_CAA11773.1	83.57
**AORI_1494**	*oxyD*	Cytochrome P450	lcl|Y16952.3_cdsid_CAC48370.1	85.35
**AORI_1495**	*hmaS*	4-hydroxymandelate synthase	lcl|AJ223998.1_cdsid_CAA11761.1	75.56
**AORI_1496**	*hmo*	4-hydroxymandelate oxidase	lcl|Y16952.3_cdsid_CAC48372.1	85.24
**AORI_1497**		Antipoter	lcl|Y16952.3_cdsid_CAC48373.1	80
**AORI_1498**	*vcaA*	Oxidase	lcl|Y16952.3_cdsid_CAC48374.1	88.04
**AORI_1499**	*vcaE*	Reductase	lcl|Y16952.3_cdsid_CAC48375.1	84.21
**AORI_1500**	*vcaB*	Aminotransferase	lcl|AJ223998.1_cdsid_CAA11782.1	88.62
**AORI_1501**	*vcaD*	Epimerase	lcl|Y16952.3_cdsid_CAC48377.1	85.57
**AORI_1502**	*dpgA*	Polyketide synthase	lcl|AJ223998.1_cdsid_CAA11765.1	92.9
**AORI_1503**	*dpgB*	Isomerase	lcl|Y16952.3_cdsid_CAC48379.1	75
**AORI_1504**	*dpgC*	Thioesterase	lcl|Y16952.3_cdsid_CAC48380.1	83.25
**AORI_1505**	*dpgD*	Dehydration protein	lcl|Y16952.3_cdsid_CAC48381.1	86.49

Note: “**-**” represents no orthologous gene was found in *bal* (Y16952.3) or *cep* (AL078635.1, AJ223999.1 and AJ223998.1) clusters.

**Figure 5:**
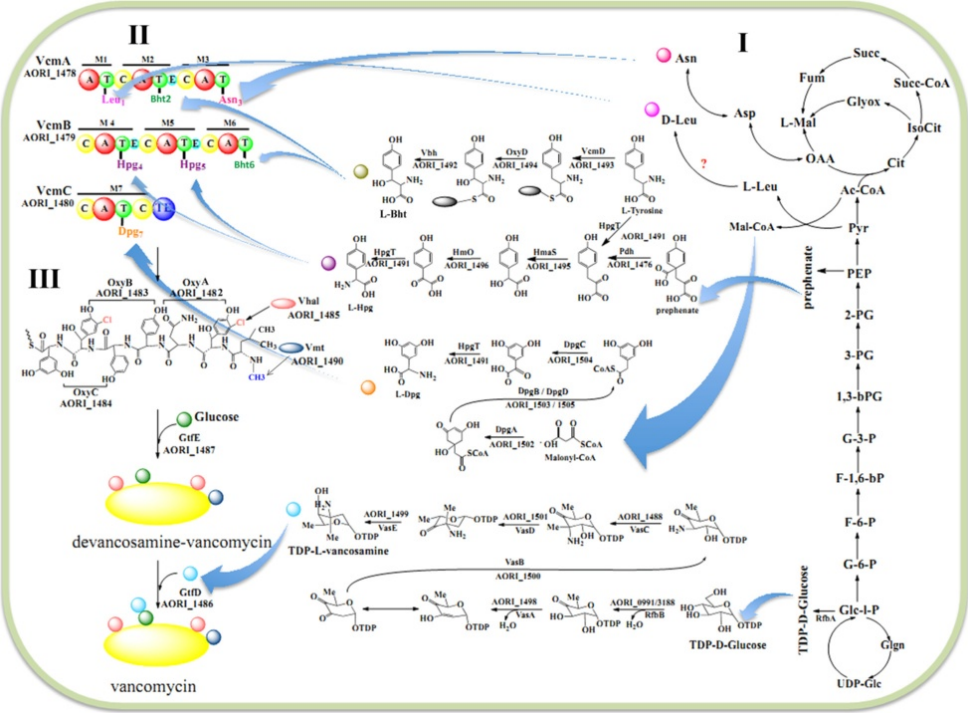
**Metabolic pathway of vancomycin biosynthesis.** Three steps are involved in the biosynthesis of vancomycin, and the related functional genes in and outside of the *vcm* cluster were mapped. **I)** The biosynthesis of its amino-acid precursors (right of the panel). Non-ribosomal peptide synthetase VcmD (AORI_1493) catalyzes free tyrosines to form tyrosyl-S-enzyme, which is hydroxylated by OxyD (AORI_1494) and then release as βHt by the action of Vhp (AORI_1492). Genes of *pdh/hpgT/hmaS/hmO* (AORI_1476, AORI_1491, AORI_1495-1496) are responsible for Hpg synthesis from prephenate, and *dpgA/B/C/D* (AORI_1502-AORI_1505) are responsible for Dpg synthesis using malonyl-CoA as the starting unit. **II)** The modified amino acids are assembled to form linear heptapeptide by NRPSs (VcmABC, AORI_1478-1480) with seven modules (M1-M7, upper left of the panel). A, adenylation domain; C, condensation domain; E, epimerization domain; T, thiolation domain; TE, thioesterase domain. **III)** The post-modifications of the linear heptapeptide (down the left side of the panel) include cyclization (*oxyA/B/C*, AORI_1482-AORI_1484), halogenation (*vhal*, AORI_1485), methylation (*vmt*, AORI_1490), and glycosylation (*gtfDE*, AORI_1486-AORI_1487). Finally, vancomycin is generated.

Similar to the biosynthesis of balhimycin and chloroeremomycin, the biosynthesis of vancomycin includes three steps [17]. The related functional genes inside and outside of the *vcm* cluster were mapped to the *A. orientalis* genome (Figure 5). First, seven amino-acid precursors, including one leucine, one asparagine, two β-hydroxytyrosine (L-βHt), two 4-hydroxyphenylglycine (L-Hpg) and one 3, 5-dihydroxyphenylglycine (L-Dpg), need to be synthesized. Genes encoding the enzymes responsible for the biosynthesis of three non-protein amino acids were identified in the genome, *i.e.*, AORI_1492-AORI_1494 for L-βHt, AORI_1476, AORI_1491, AORI_1495-AORI_1496 for L-Hpg, and AORI_1502-AORI_1505 for L-Dpg.

Second, the seven precursor amino acids are assembled to form a heptapeptide backbone, which are catalyzed by the NRPSs VcmA (AORI_1478), VcmB (AORI_1479), and VcmC (AORI_1480). These three giant enzymes contain seven modules (M1-M7) with 24 domains that function in the selection, activation, condensation and epimerization of the amino-acid substrates [17]. In M2, M4, and M5, there are three epimerases (E domain) that convert L-βHt_2_, L-Hpg_4_, and L-Hpg_5_ into the corresponding D type amino acids. The N-terminal amino acid of vancomycin is D-methylleucine [47]. However, neither an epimerase nor a dual condensation/epimerization domain [48, 49] was observed in M1 or the adjacent C domain in M2. Rausch *et al.* conjectured that a racemase outside the *vcm* cluster might be responsible for the conversion of L-leucine into D-leucine, which can be incorporated directly into the glycopeptides [48]. Throughout the whole genome, there are 11 genes that potentially encode racemases, including six amino-acid racemases, three CoA racemases, and two mandelate racemases (see Additional file 1: Table S6). The recent genomic analysis of *Vibrio cholera* identified a novel PLP-dependent amino-acid racemase (vc1312) that was proven to be necessary and sufficient for the synthesis of the unusual D-amino acids, including D-leucine [50]. With vc1312 as the query sequence, we used the BLASTP program to search throughout the whole genome of *A. orientalis*. The results revealed one protein, annotated as an amino-acid racemase (AORI_0725), which has 28% amino-acid identity (48% positive) with vc1312 and that may function in D-leucine conversion. Further experimental proof is required to confirm its involvement in vancomycin synthesis.

The last step is the post-assembly modifications of the heptapeptide backbone, including its cyclization, halogenation, methylation and glycosylation. Based on their corresponding genes in the *cep* and *bal* clusters [7, 8], the functions of the modification genes in the *vcm* cluster were annotated (Table 2). The *oxyA/B/C* (AORI*_*1482-1484) genes likely encode three P450 monooxygenases that are responsible for closing the linear peptide to form the heptapeptide ring [19, 51]. Adjacent to them, AORI_1485 (*vhal*) is predicted to encode a halogenase, showing 94% amino-acid sequence identity with that encoded by the *bhaA* in *bal*[9], which chlorinates the βHt residues. However, the exact timing of the chlorination is unknown, although it was proposed to occur before the oxidative couplings [52]. The methylation of D-leucine on the α-NH_2_ is catalyzed by a methyltransferase, which has been functionally characterized in the *cep* cluster [14]. Its orthologous protein in the *vcm* cluster was found and annotated as Vmt (AORI_1490). Glycosylation is the last of the modifications and the functional glycosyltransferases for vancomycin biosynthesis have been well-characterized biochemically [10, 15]. GtfE (AORI_1487) is responsible for the addition of the first TDP-glucose moiety to the 4'-hydroxyl group of amino acid Hpg_4_, and the other glycosyltransferase GtfD (AORI_1486) adds the second TDP-L-β-vancosamine moiety to the 2'-hydroxyl group of a glucose residue. AORI_1487 shows the highest amino acid sequence similarity to BgtfB encoded by *bal* (81%) and GtfB encoded by *cep* (81%), whereas AORI_1486 shows the highest similarity to BgtfC encoded by *bal* (70%) and GtfC encoded by *cep* (69%). No glycosyltransferase corresponding to BgtfA or GtfA, which add 4-*epi*vancosamine to the amino-acid residue of βHt_6_ in *bal* or *cep*, was found in the genome of *A. orientalis*. Therefore, there is no *epi*-vancosamine moiety present in vancomycin (Figure 1).

**Figure 6:**
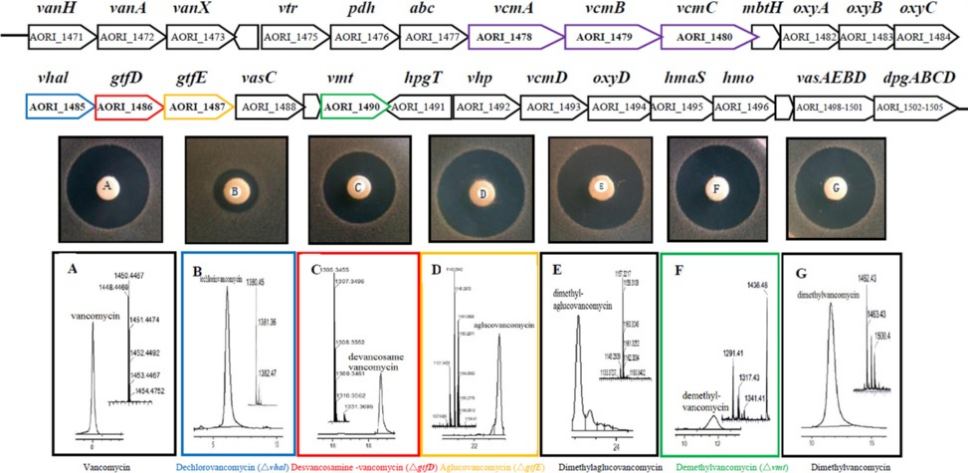
**Functional characterization and verification of the modification genes in the** ***vcm*** **cluster.** The 64-kb *vcm* cluster is illustrated in detail. AORI_1490 (*vmt*), AORI_1486 (*gtfD*), AORI_1487 (*gtfE*), and AORI_1485 (*vhal*) were replaced in-frame by selection markers, and AORI_1490 was overexpressed *in vitro* using demethylvancomycin/aglucovancomycin as the substrate. The vancomycin standards **(A)** and the corresponding variants obtained by isolation from mutant strains or the *in vitro* treatments were detected by HPLC-MS: **(B)** dechlorovancomycin, **(C)** desvancosamine vancomycin, **(D)** aglucovancomycin, **(E)** dimethylaglucovancomycin, **(F)** demethylvancomycin, and **(G)** dimethylvancomycin. The structural formulae of the variants are shown in the table of Figure 1. A mass of 20 μg of each compound was used to assay its activity against MRSA, and the picture is representative of three independent experiments.

To characterize the *in vivo* functions of the predicted halogenase, the putative methyltransferase and the glycosyltransferases encoded by the *vcm* cluster, in-frame monogenic mutants of AORI_1485 (*vhal*), AORI_1490 (*vmt*), AORI_1486 (*gtfD*), and AORI_1487 (*gtfE*) are successfully constructed using a homologous recombination method similar to the PCR-targeting system (Methods). Various types of vancomycin derivatives, *i.e.*, dechlorovancomycin, demethylvancomycin, desvancosamine vancomycin, and aglucovancomycin, which accumulated in the corresponding mutant cultures, were collected and their structures were confirmed by high-performance liquid chromatography-mass spectrometry (HPLC-MS) (Figure 6). Based on the results from the zone of inhibition test, desvancosamine vancomycin (Figure 6C), particularly dechlorovancomycin (Figure 6B), showed a lower bioactivity relative to that of vancomycin, whereas aglucovancomycin (Figure 6D) showed a slightly higher bioactivity than that of vancomycin. The bioactivity of demethylvancomycin (Figure 6F) was comparable to that of vancomycin. In addition, using demethylvancomycin or aglucovancomycin as the substrate, dimethylvancomycin (Figure 6G) or dimethylaglucovancomycin (Figure 6E) were generated *in vitro* catalyzed by the heterogeneously expressed methyltransferase AORI_1490. Their molecular weights were also confirmed by the HPLC-MS spectrum, and the positions of the two methyl groups on the N terminus of leucine (Figure 1) were further examined using nuclear magnetic resonance (NMR) (Additional file 1: Table S7, Additional file 1: Table S8). Compared with that of vancomycin (Figure 6A), dimethylvancomycin showed a comparable antibacterial activity. Although dimethylaglucovancomycin (Figure 6E) is a novel compound, its activity was also similar to that of aglucovancomycin. Taken together, both methylation and demethylation do not affect the *in vitro* antibacterial activity of vancomycin or its derivatives. For glycosylation, despite aglucovancomycin showing a slightly higher bioactivity than that of vancomycin *in vitro*, the *in vivo* activity was five-fold lower than that of vancomycin [53], indicating that the sugar moiety may play an important role in imparting enhanced pharmacokinetic properties.

With the exception of dimethylaglucovancomycin, nearly all of the vancomycin derivatives mentioned above have been isolated naturally and their antibacterial activities reported [53] and our results are in agreement with these previous findings. However, this study provides, for the first time, *in vivo* functional characterization of the predicted halogenase, the putative methyltransferase and the biochemically-characterized glycosyltransferases in *A. orientalis*, along with a systemic analysis of the distinct bioactivities of different vancomycin variants. These *in vivo* analyses not only demonstrated that the *vcm* encoded halogenase and methyltransferase are functionally equivalent to those encoded in the *bal* and *cep* clusters, but also inferred that the modifications of halogenation, methylation, and glycosylation are not conducted exactly in series [17], because the vancomycin variants produced by each mutant were only deficient in their corresponding modification principally (the only exception is aglucovancomycin of the *gtfE* mutant, from which both the glycosyl residues were absent)*.* In other words, the tailoring enzymes (except GtfD) are not very specific, but have broad substrate spectra *in vivo*.

## Conclusions

The genome of *A. orientalis* HCCB10007 is the first complete sequence for the bacteria that synthesize the vancomycin group antibiotics. Compared with the phylogenetic closely related rifamycin-producing strain *A. mediterranei*, *A. orientalis* has a relatively smaller chromosome and a more compact genomic organization. Their different configurations revealed possible chromosomal recombination events representing genomic plasticity related to either vancomycin biosynthesis or rifamycin biosynthesis. By comparison with other actinomycete genomes, the common features of the *Amycolatopsis* genomes and molecular chemotaxonomic **c**haracteristics (MCCs) representing the phenotypes of phospholipid and menaquinone for this genus were further identified and developed. In addition, the knockout of genes encoding the tailoring enzymes in *A. orientalis* was achieved, and the functions of the predicted halogenase and methyltransferase annotated in the *vcm* cluster were characterized for the first time. The data provided by this study may facilitate the development of novel lead compounds for drug development, either through a combinatorial biosynthesis approach employing enzymes with newly engineered modification activities, or using different vancomycin derivatives as the starting chemical moieties.

## Methods

### Genome sequencing and assembly

*A orientalis* strain HCCB10007 was deposited in the Institute of Microbiology of Chinese Academy of Sciences and designated CGMCC No. 6023. A traditional whole genome shotgun strategy using the Roche 454 GS FLX Titanium System was applied to sequence HCCB10007’s genome. In total, 53 contigs with 8.9-Mb length were assembled from 561,423 reads (average length of 408 bp) by the Newbler Program (version 2.3) of the 454 suite package. The relationships between the contigs were determined by referring to the *A. mediterranei* genome or using the ContigScape plugin [54], and the remaining gaps were filled using a multiplex PCR strategy. The final sequence assembly was performed using the phred/phrap/consed package (http://www.phrap.org/phredphrapconsed.html). Sanger-based sequencing was employed to facilitate the gap closure and to amend the low-quality regions (score < 60). Finally, a consensus sequence containing 8,948,591 bp (with an estimated error rate of less than 0.5 per 100,000 bases) that provided 25.6-fold coverage was acquired.

### Genome annotation and analysis

Putative protein-coding sequences were predicted based on the results from both Glimmer and Genemark. The BLASTP results obtained from the KEGG, NR, and CDD databases were used to annotate the CDSs and manual correction was also implemented. The tRNA genes were predicted directly with tRNAscan-SE v1.23. Essential genes were defined as those encoding proteins functioning in cell division, replication, transcription, translation, and amino-acid metabolism, with reference to the Clusters of Orthologous Groups (COGs) Database. Unless otherwise stated, the orthologous proteins between *A. orientalis* HCCB10007 and other related species were defined by reciprocal BLASTP under the following conditions: minimum 30% identity and 30% length diversity. The coding density of all of the genes was defined as the ratio of the protein-coding sequences (CDSs) length to the total genomic length, whereas the coding density of essential genes (or orthologs) was defined as the ratio of sequence length of the essential genes (or orthologs) to the total CDSs in a corresponding non-overlapping sliding window. Statistical comparisons between core *vs*. non-core and quasi-core *vs*. non-core were estimated as *P*-values calculated by the grouped t test method with the statistical programming language R, employing two window sizes for analyses (except for the case of comparing the coding density of essential genes between quasi-core *vs*. non-core, *P*-values shown in the text used the 90-kb sliding window size instead of 45-kb). The MUMmer 3.0 Project was used to analyze the genome-wide co-linearity between *A. orientalis* HCCB10007 and *A. mediterranei*. To characterize the molecular chemotaxonomic characteristics (MCCs), the biosynthetic pathways of arabinose, glycine, diaminopimelic acids, mycolic acids, phospholipids, and menaquinones in *A. orientalis* and other actinomycetes were analyzed on http://www.kegg.jp/kegg/pathway.html. Literature searching, sequence alignment, domain comparison, and/or phylogenetic analysis were used to further identify the critical genes that determined the existence, conformation, and chain lengths of compounds. All of the BLASTP analyses conducted with the MCCs used a threshold E value of 1e-3. The neighbor joining (NJ) method of the MEGA 5.0 package was used to construct phylogenetic trees based on the 16S rRNA, Isp, and MurE sequences, and the reliability of each branch was tested by 1,000 bootstrap replications. For the Isp sequences, an additional maximum parsimony (MP) method was used to obtain a more robust tree topology. The SBSPKS [45] and/or NRPSDB [46] databases were used to predict the probable substrates or products for secondary metabolite biosynthetic gene clusters.

### Construction of monogenic mutations of AORI_1485 (*vhal*), AORI_1490 (*vmt*), AORI_1486 (*gtfD*) and AORI_1487 (*gtfE*) in *A. orientalis*

A homologous recombination method similar to the PCR-targeting system applied in *Streptomyces* was used to mutate the *vhal*, *vmt*, *gtfD*, and *gtfE* genes in *A. orientalis* HCCB10007. First, a cosmid library of *A. orientalis* genomic DNA (containing inserts of about 40Kb in length) was constructed using SuperCos 1 Cosmid Vector Kit (Agilent Technologies, Inc.). The cosmid clone XL0311, which contained the AORI_1485, AORI_1490, AORI_1486, and AORI_1487 genes, was selected by Southern blotting and further used to knockout *vhal*, *vmt*, *gtfD*, and *gtfE*, individually. All of the target genes in XL0311 were replaced precisely with the apramycin-resistance gene, using two long recombinational primers (39 nt). The cosmids with mutated target genes were introduced into *E. coli* BW25113/pIJ790 (λ RED recombination plasmid) and then conjugated into the *A. orientalis* recipients. The correct exconjugants/knockout clones were selected based on their apramycin resistance (50 μg/ml) on MS medium (mannose 20 g/L, soybean flour 20 g/L, agar 20 g/L, and 10 mmol/L MgCl_2_) supplemented with nalidixic acid (20 μg/ml) for counter selection. The genes inactivated by homologous recombination were further confirmed by PCR (for primers, please see Additional file 1: Supplementary Materials and Methods).

### Vmt expression, purification, and *in vitro* modification assay

The *vmt* gene from the *A. orientalis* HCCB10007 genomic DNA was cloned into a pET30a vector and transformed into BL21 (DE3) cells. The expression of His_6_-tagged Vmt was induced by 1 mM isopropyl-1-thio-β-D-galactoside (IPTG) at 30°C for 4 h, and then nickel-nitrilotriacetic acid (Ni-NTA) affinity chromatography (Qiagen, Valencia, CA, USA) was used to purify the protein. The *in vitro* methylation modification system contained 2 mM (*S*)-adenosyl-L-methionine (Sigma-Aldrich Canada), 10 mM His_6_-Vmt (3 mg), 500 mM substrate (demethylvancomycin or aglucovancomycin), and 50 mM Tris-HCl (pH 7.5) in a total volume of 1 ml. The reaction was conducted at 25°C for 24 h. The reaction was stopped by the addition of an equal volume of cold methanol, incubated at –20°C for 20 min, and centrifuged for 5 min at 10,000 × g. HPLC-MS was used to analyze the supernatant.

### HPLC-MS analyses

The vancomycin derivatives were prepared from ultrasonically lysed suspensions of the culture pellets, and the cell debris was removed by centrifugation and filtration. HPLC-Q-TOF-MS (Waters Micromass Q-TOF Premier Mass Spectrometer) analysis was then used to identify the derivates. HPLC was performed at 40°C using an ACQUITY HPLC BEH C18 column (100 mm × 2.1 mm, i.d.: 1.7 μm; Waters Corp., Milford, USA) equipped with an ACQUITY HPLC VanGuard PreColumn (5 mm × 2.1 mm, i.d.: 1.7 μm; Waters Corp., Milford, MA, USA). Solvent A (0.05% TFA in water) and solvent B (0.05% TFA in acetonitrile) were used as the mobile phase, with a flow rate of 0.4 ml min^-1^. The following gradient was used: t = 0 min: 5% B; t = 2.2 min: 15% B; t = 4.5 min: 30% B; t = 12.5 min: 99% B. The mass spectrometer detected all of the samples at a wavelength of 240 nm.

### Zone of inhibition test

This test was conducted using *Staphylococcus aureus* cultured in LB medium as indicator cells. The soft top agar of the test agar plate consisted of 10 gL^-1^ tryptone extract, 5 gL^-1^ yeast extract, 5 gL^-1^ NaCl, and 16 gL^-1^ agar, and the indicator cells were added into the soft agar at a final concentration of 10^6^ cfu/ml. Then, 20 μg of each vancomycin variant was carefully dropped onto the drug-sensitive slips. The slips were then placed on the center of the agar plates. Observations were made after 20 hours at 37°C. Three independent experiments were performed and the representative pictures were chosen for the figure.

### Nucleotide sequence accession numbers

The nucleotide sequences of the chromosome and plasmid were deposited in the GenBank database under accession numbers [CP003410] and [CP003411], respectively.

### Availability of supporting data

All the supporting data named as “Additional files for the *Amycolatopsis orientalis* genome paper”, were deposited in an open access repository of CHGC (Chinese National Human Genome Center at Shanghai) database. Please refer to http://chgc.sh.cn/ch/Ao.html. The phylogenetic trees based on the 16S rRNA, Isp, and MurE sequences were deposited in Treebase database (http://www.treebase.org). Please refer to http://purl.org/phylo/treebase/phylows/study/TB2:S15601.

## Electronic supplementary material

Additional file 1: Figure S1: (A) Phylogeny tree based on 16S ribosome RNA of selected actinobacteria and other related species. (B) Comparative analyses of the orthologs between different actinomycete genomes. **Table S1.** Comparative analysis of the 16S ribosome RNAs between and in *A. orientalis* and *A. mediterranei* genomes. **Table S2.** The *P*-values derived from grouped t test for the coding densities of orthologs or essential genes comparing the core (or R1, or R2) region against the non-core regions under the conditions of different sliding window sizes. **Table S3.** Enzymes in different actinomycetes involved in the pathway of incorporating arabinose into the cell wall. **Table S4.** Genes characterized in different actinomycetes responsible for recruiting glycine residues crossbridging to the peptidoglycan lateral chains. **Figure S2.** Pylogenetic analyses of MurE in actinomycetes. **Figure S3.** Genetic organization of the *fadD-pks-accD* and *fas-I* gene clusters in 20 selected actinobacterial genomes. **Figure S4.** phylogenetic analysis of isoprenyl diphosphate synthases from type strains of actinomycetes using the MP method. **Table S5.** Orthologs of secondary metabolite genes in *A.orientalis* HCCB10007 genome compared to the NCBI database. **Figure S5.** The reverse-transcription PCR of RNA isolated from different cultures. **Table S6.** Genes encoded for racemases in *A.orientalis* HCCB10007 genome. **Table S7.** NMR spectroscopic data for dimethylvancomycin. **Table S8.** NMR spectroscopic data for dimethylaglucovancomycin. (PDF 952 KB)
