# Supplementary material for: Complete genome sequence and comparative genomic analyses of the vancomycin-producing Amycolatopsis orientalis
Source: BMC Genomics. 2014 May 13;15(1):363. doi: 10.1186/1471-2164-15-363 (PMC4048454; doi:10.1186/1471-2164-15-363)
Supplement: Supplementary file 1 — Additional file 1: Figure S1: (A) Phylogeny tree based on 16S ribosome RNA of selected actinobacteria and other related species. (B) Comparative analyses of the orthologs between different actinomycete genomes. Table S1. Comparative analysis of the 16S ribosome RNAs between and in A. orientalis and A. mediterranei genomes. Table S2. The P-values derived from grouped t test for the coding densities of orthologs or essential genes comparing the core (or R1, or R2) region against the non-core regions under the conditions of different sliding window sizes. Table S3. Enzymes in different actinomycetes involved in the pathway of incorporating arabinose into the cell wall. Table S4. Genes characterized in different actinomycetes responsible for recruiting glycine residues crossbridging to the peptidoglycan lateral chains. Figure S2. Pylogenetic analyses of MurE in actinomycetes. Figure S3. Genetic organization of the fadD-pks-accD and fas-I gene clusters in 20 selected actinobacterial genomes. Figure S4. phylogenetic analysis of isoprenyl diphosphate synthases from type strains of actinomycetes using the MP method. Table S5. Orthologs of secondary metabolite genes in A.orientalis HCCB10007 genome compared to the NCBI database. Figure S5. The reverse-transcription PCR of RNA isolated from different cultures. Table S6. Genes encoded for racemases in A.orientalis HCCB10007 genome. Table S7. NMR spectroscopic data for dimethylvancomycin. Table S8. NMR spectroscopic data for dimethylaglucovancomycin. (PDF 952 KB) [file 12864_2013_6095_MOESM1_ESM.pdf]

Additional files

Complete genome sequence and comparative genomic analyses of the vancomycin-producing *Amycolatopsis orientalis*

Li Xu[1,2]*, He Huang[3]*, Wei Wei[2]*, Yi Zhong[3#], Biao Tang[4], Hua Yuan[3], Li Zhu[2], Weiyi Huang[1], Mei Ge[2], Shen Yang[3], Huajun Zheng[5], Weihong Jiang[3 §], Daijie Chen[2,6 §], Guo-Ping Zhao[3,4,5,7 §], Wei Zhao[3,4,8,9 §]

*These authors contributed equally to this work

[#]Current address: Computational Biology Center, Memorial Sloan Kettering Cancer Center, New York, NY 10065, United States.

[§]Corresponding authors: W.Z.: 181zhao@163.com; G-P.Z.: gpzhao@sibs.ac.cn; D.C.: hccb001@163.com; W.J.: whjiang@sibs.ac.cn

Supplementary *Materials and Methods*

Total DNA extraction

Spores of *Amycolatopsis orientalis* HCCB10007 were inoculated into a 250 ml-baffled flask containing 25 ml TSB (Tryptone Soya Broth, 30g L^{-1}) medium and cultivated at 28 ℃, 220 rpm for 32 h. The cells were harvested and the genomic DNA was extracted directly from the expanded culture for 454 sequencing.

Scanning electron microscopy

A.orientalis HCCB10007 was cultured on Gause medium (amidulin 20 g L^{-1}, NaCl 0.5 g L^{-1}, K_2HPO_4 0.5 g L^{-1}, KNO_3 1.0 g L^{-1}, $MgSO_4{\cdot}7H_2O$ 0.5 g L^{-1}, $FeSO_4{\cdot}7H_2O$ 0.01 g L^{-1} agar 20 g L^{-1}) covered with cellophane, the cells on which were fixed with fresh 2% glutaraldehyde (pH 7.2) and 1% osmium tetroxide after 1-day or 3-days incubation at 28 ℃. After dehydration, ethanol was replaced by amyl acetate. The samples were then dried in HCP-2 (Hitachi, Tokyo, Japan) using the supercritical drying method, subsequently coated with gold by Fine Coater JFC-1600 (Jeol, Tokyo, Japan), and then examined with a JSM-6360LV scanning electron microscopy (SEM; Jeol).

RNA isolation and reverse transcription PCR

A. orientalis HCCB10007 spores were inoculated into a 250 ml-baffled flask containing 25 ml seed medium and cultivated at 28 ℃ and 220 rpm for 44 h, which then transferred to the fermentation medium F1 or the nutrient medium F5 at 28 ℃ and 220 rpm for 48 h. The seed medium contains glycerol (20g L^{-1}), amidulin (40 g L^{-1}), soybean flour (20 g L^{-1}), glucose (15 g L^{-1}), KNO_3 (6 g L^{-1}), KH_2PO_4 (0.2 g L^{-1}), $MgCl_2{\cdot}6H_2O$ (0.4 g L^{-1}). The fermentation medium F1 contains glycerol (20 g L^{-1}), soybean flour (20 g L^{-1}), KNO_3 (6 g L^{-1}), KH_2PO_4 (0.2 g L^{-1}), $MgCl_2.6H_2O$ (0.4 g L^{-1}),

$CaCO_3$ (3 g L^{-1}), while the composition of nutrient medium F5 is the same as that of the F1 except that yeast extract is instead of soybean flour.

A. orientalis HCCB10007 total RNA was isolated from the F1 or F5 medium according to the description for streptomyces elsewhere. The quantity of RNA was examined by denaturing formamide agarose gel electrophoresis. One microgram of RNA was used to synthesize cDNA in a total volume of 10 μl using the SuperScript™ III Reverse Transcriptase (Invitrogen, California, USA). After incubation of 1 h at 50 ℃, 1μl of the cDNA reaction mixture was used as the template for the following PCR.

The culture conditions for *A. orientalis* HCCB10007 and other mutant strains

The culture conditions were the same for *A. orientalis* and all the mutants. Spores were inoculated into a 250 ml-baffled flask containing 25 ml seed medium and cultivated at 28 ℃ and 220 rpm for 48 h, which then transferred (8% inoculum dose) to the fermentation medium at 28 ℃ and 220 rpm for 120 h.

The seed medium contains glycerol (20g L^{-1}), amidulin (40 g L^{-1}), soybean flour (20 g L^{-1}), glucose (15 g L^{-1}), KNO_3 (6 g L^{-1}), KH_2PO_4 (0.2 g L^{-1}), $MgCl_2 \cdot 6H_2O$ (0.4 g L^{-1}).

The fermentation medium (PH6.5) contains glycerol (20 g L^{-1}), soybean flour (20 g L^{-1}), KNO_3 (6 g L^{-1}), KH_2PO_4 (0.2 g L^{-1}), $MgCl_2.6H_2O$ (0.4 g L^{-1}), $CaCO_3$ (3 g L^{-1}).

The nuclear magnetic resonance (NMR) analysis of dimethylvancomycin and dimethylaglucovancomycin

For dimethylvancomycin (40mg ml^{-1}), ^{1}H-NMR was measured at 500 MHz in D_2O, and ^{13}C-NMR was measured at 125 MHz in D_2O. For dimethylaglucovancomycin (40mg ml^{-1}), ^{1}H-NMR was measured at 400 MHz in DMSO-d_6, and ^{13}C-NMR was measured at 100 MHz in DMSO-d_6. The ^{1}H and ^{13}C

NMR spectra were recorded on Bruker Avance-400 spectrometer with TMS as an internal standard.

Primers used for RT-PCR

**Cluster primers**	
nrps3F	CAGATTCACCGGGACCGTCG
nrps3R	TCGGCCTCCTTCGCGTACA
nrps4F	ACCGTCGGGTGGTTCACC
nrps4R	ACCAGTCGGTCGCTTCGGG
nrps7F	GCCGTGGCGGTCGATCTGGA
nrps7R	TCGCTCGCGTGGTCACCCTG
n_p1F	GCAACGTCGGGATCGCGCT
n_p1R	ACGTAGGCGAGCCCGATCCC
n_p2F	ATTCAAGCAGCCGGTCCAGG
n_p2R	ACCTGCTGGTCGGATTGGCC
n_p3F	GCCACGGCGGTGGTGAACTA
n_p3R	ACCACACGTCCGTCCGCCAT
pks3F	GGGAAACCGGCGCACGAGA
pks3R	ATCAGCAACACCGGCCCGG
pks5F	GCCGAAGAAGGCGAGGAGGTCG
pks5R	ACGCTGGTGCCGGAGCACAC
pks9F	CTCGCCCCAGTTCATCAACG
pks9R	CAACCGGGTGATCCTGTTCG
vcmF	GCCGACTACCTCGCGCAGAT
vcmR	CCAGAGATTGCGCATGACCG
ecoF	TCCTGGTGGCCCACTCCTCT
ecoR	TGATCCCCCTCGACGTCCAC

Primers used for amplifying the DNA sequences of unique gene in different strains

ForAORI_2733-C-F:	TGGACGCACTACTCGATCGC
ForAORI_2733-C-R:	TACTGGCTGCACCACCACAT

Primers used for gene inactivation and checking

Gene	Primers
vmtF	GGGCCTGAGACGAATGTCGACCTGGAGGTTGTCCTGATGATTCCGGGGATCCGTCGACC
vmtR	TGATCGAGGAGGGCGTGAGCAGGCTCGCCGCGCTCATCATGTAGGCTGGAGCTGCTTC
vmt-C-F	CGAACTCCCGGAAGGATTCC
vmt-C-R	ATGAGCTCAGCCAATCGA
VhalF	GCGCCGTGGCTGTGCGGTGAGCGGGGAAGGACCATCATGATTCCGGGGATCCGTCGACC
vhalR	CGAACAGGGCCGGTGTCGTGGCCGCCGAAAAAACGGTCATGTAGGCTGGAGCTGCTTC
vhal-C-F	AAGGTACGGTCACCGCAGTG
vhal-C-R	ATGAGCTCAGCCAATCGA
gtfDF	TGCGCCGGTGGTCGAGCACGAAACAGGGGTAGCGAAATGATTCCGGGGATCCGTCGACC
gtfDR	TTCTCCGGTCCTGGCCGGCGTCCGGTCGCTGATGGTTCATGTAGGCTGGAGCTGCTTC
gtfD-C-F	CCGTCGACAAGATGGCGAC
gtfD-C-R	ATGAGCTCAGCCAATCGA
gtfEF	CGCCGGCCAGGACCGGAGAAAACGGGGAAATACGTGATGATTCCGGGGATCCGTCGACC
gtfER	GTGGTCGACATGCGGTCTCCTCTGTACTGGTGATGATCATGTAGGCTGGAGCTGCTTC
gtfE-C-F	GGAACACCGACCAGCCGTAC
gtfE-C-R	ATGAGCTCAGCCAATCGA

*The nucleotides underlined are primers for antibiotics resistance gene amplification.

Primers used for Vmt expression

Primers	
vmt-E-F	CATATGACCGATCAACTGGACCGC
vmt-E-R	AAGCTTCTTCGCGTCTGCCGTGAC

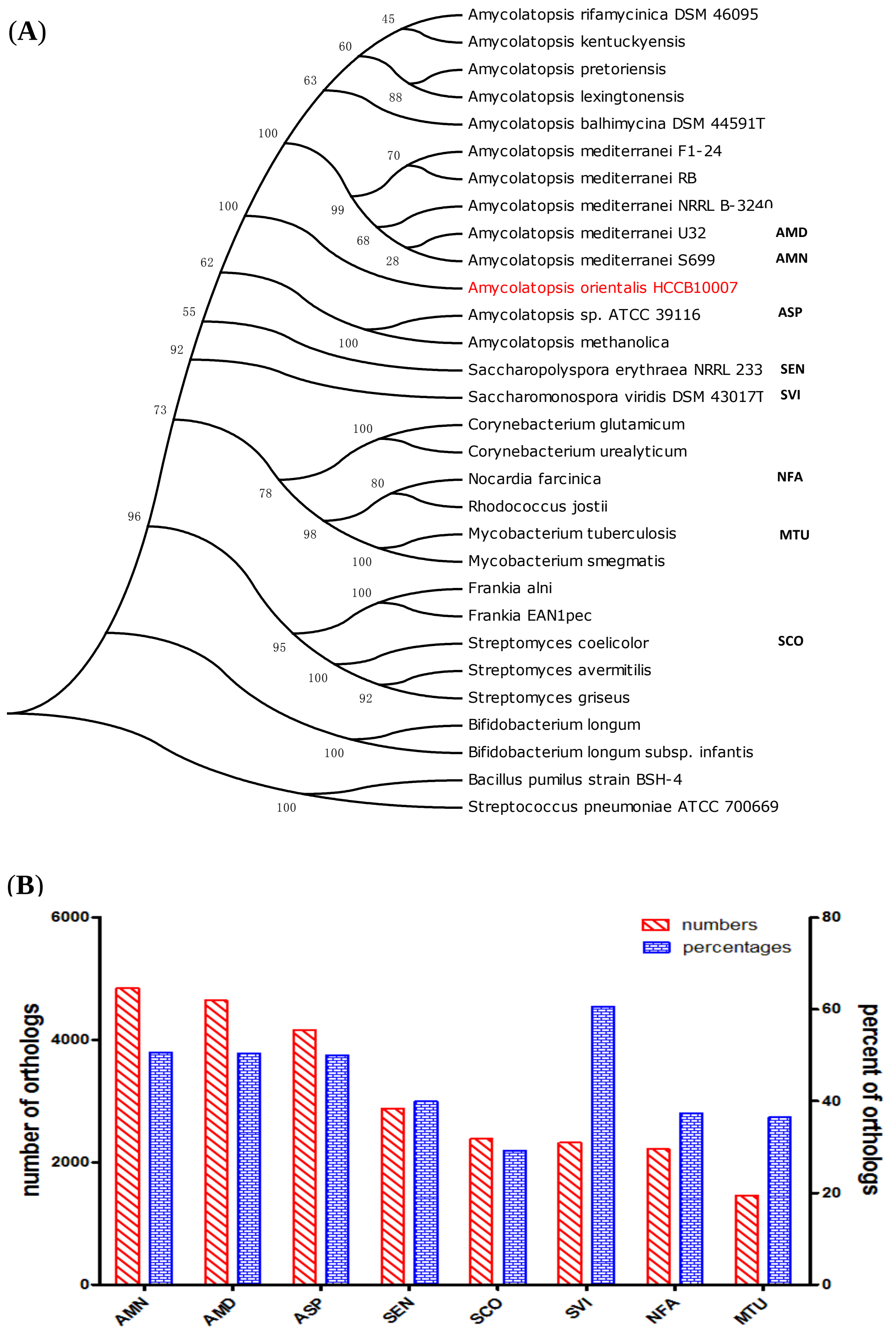

Figure S1. (A) Phylogeny tree based on 16S ribosome RNA of selected actinobacteria and other related species. (B) Comparative analyses of the orthologs between different actinomycete genomes. The orthologs of *A. orientalis* are shared with *A. mediterranei* S699 (AMN), *A. mediterranei* U32 (AMD), *A. sp* (ASP), *S. erythraea* (SEN), *S. coelicolor* (SCO), *S. viridis* (SVI), *N. farcinica* (NFA),

and *M. tuberculosis* (MTU). The red bars represent the number of orthologs (left vertical axis), and the blue bars represent the percentages of orthologs in each species (right vertical axis). All the sequences (including the 16S ribosome RNA) were obtained from NCBI at http://www.ncbi.nlm.nih.gov/.

Table S1. The comparison of 16S rRNA between and in *A. orientalis* (four sequences: #1, #2, #3, #4) and *A. mediterranei* (four sequences: #a, #b, #c, #d) genomes

			Serial number	#1	#2	#3	#4	#a	#b	#c	#d
			location	core	core	quasi-core	core	core	core	quasi-core	quasi-core
			Transcriptional direction	+	+	-	-	+	+	-	-
Serial number	location	Transcriptional direction		AORI_R016	AORI_R022	AORI_R041	AORI_R045	AMED_R16	AMED_R25	AMED_R39	AMED_R49
#a	core	+	AMED_R16	97.20	97.20	97.20	97.20	100.00			
#b	core	+	AMED_R25	97.27	97.20	97.20	97.20	99.93	100.00		
#c	quasi-core	-	AMED_R39	97.13	97.13	97.13	97.13	99.93	99.86	100.00	
#d	quasi-core	-	AMED_R49	97.13	97.13	97.13	97.13	99.93	99.86	100.00	100.00
#1	core	+	AORI_R016	100.00							
#2	core	+	AORI_R022	98.91	100.00						
#3	quasi-core	-	AORI_R041	99.04	98.63	100.00					
#4	core	-	AORI_R045	99.04	98.63	100.00	100.00				

Table S2: The *P*-values derived from grouped t test for the coding densities of orthologs or essential genes comparing the core (or R1, or R2) region against the non-core regions under the conditions of different sliding window sizes*.

Gene category for comparison	window size	*P* values for the comparisons		
		core vs non-core	R1 vs non-core	R2 vs non-core
orthologs	90kb	4.34*1e-10 (sample: 63 vs 30)	0.011 (sample: 3 vs 30)	2.2*6e-05 (sample: 3 vs 30)
essential genes	90kb	5.1*1e-04 (sample: 63 vs 30)	0.14 (sample: 3 vs 30)	0.17 (sample: 3 vs 30)
essential genes	45kb	8.5*1e-05 (sample: 127 vs 61)	0.051 (sample: 5 vs 61)	0.046 (sample: 5 vs 61)

*Refer to the ***Methods*** for details.

Table S3. Enzymes in different actinomycetes involved in the pathway of incorporating arabinose into the cell wall (Note: "√", present in the microorganism; "-", absent in the microorganism)

Species \ Enzymes	Transketolase	Phosphoribosyl pyrophosphate synthetase	5-phosphoribosyl transferase	Phospholipid phosphatase	Epimerase1	Epimerase2
Nocardia farcinica IFM 10152	√	√	√	√	√	√
Mycobacterium tuberculosis H37Rv	√	√	√	√	√	√
Saccharopolyspora erythraea NRRL23338	√	√	√	√	√	√
Corynebacterium glutamicum ATCC 13032	√	√	√	√	√	√
Rhodococcus jostii RHA1	√	√	√	√	√	√
Amycolatopsis mediterranei U32	AMED_2809	AMED_8235	AMED_0226	AMED_0225	AMED_0230	AMED_0229
Amycolatopsis mediterranei S699	RAM_14275	RAM_42295	RAM_01145	RAM_01140	RAM_01165	RAM_01160
Amycolatopsis orientalis HCCB10007	AORI_2796	AORI_7026	AORI_0229	AORI_0228	AORI_0235	AORI_0234
Streptomyces. albus J1074	√	√	-	-	-	-
Streptomyces avermitilis MA-4680	√	√	-	-	-	√
Streptomyces clavuligerus ATCC 27064	√	√	-	-	-	-
Streptomyces coelicolor A3(2)	√	√	√	√	√	√
Streptomyces flavogriseus ATCC 33331	√	√	-	-	√	√
Streptomyces ghanaensis ATCC 14672	√	√	√	-	√	-
Streptomyces griseoflavus Tu4000	√	√	√	-	√	√
Streptomyces griseus subsp. *griseus* NBRC 13350	√	√	-	-	√	√
Streptomyces hygroscopicus ATCC 53653	√	√	-	-	-	-
Streptomyces lividans TK24	√	√	-	-	-	√
Streptomyces pristinaespiralis ATCC 25486	√	√	-	-	-	-
Streptomyces sp. ACTE	√	√	-	-	√	√
Streptomyces sp. C	√	√	-	-	-	-
Streptomyces sp. Mg1	√	√	-	-	-	-
Streptomyces sp. SPB74	√	√	-	-	-	-
Streptomyces sp. SPB78	√	√	-	-	-	-
Streptomyces sviceus ATCC 29083	√	√	-	-	√	√
Streptomyces viridochromogenes DSM 40736	√	√	√	-	√	√

Note: Genes (*AORI_2796, AORI_7026, AORI_0228-0229, AORI_0234-0235*) coding for enzymes involved in incorporating arabinose into the cell wall were found throughout the genome of *A. orientalis*. These genes can also be found in other arabinose-containing species in addition to *A. mediterranei*, such as *Mycobacterium tuberculosis*, *Nocardia farcinica* and *S. erythraea*, but not in the arabinose-deficiency *Streptomyces* species (except *S. coelicolor* A3(2)).

Table S4. Genes characterized in different actinomycetes responsible for recruiting glycine residues crossbridging to the peptidoglycan lateral chains (Note: "--", absent in the microorganism)

Genes / Species	***femA*** (the second and third glycine)	***femB*** (the fourth and fifth glycine)	***fmhB*** **(**the first glycine)
Staphylococcus aureus	*SA1206*	*SA1207*	*SA2057*
Streptomyces coelicolor	*SCO3593*	*SCO3904*	*SCO0602*
Nocardia farcinica	--	--	--
Saccharopolyspora erythraea	--	--	--
Amycolatopsis mediterranei	--	--	--
Amycolatopsis orientalis	--	--	--

Note: Similar to that of *A. mediterranei*, none of genes (*femA*, *femB and fmhB*) responsible for recruiting glycine residues cross-bridging to the peptidoglycan lateral chains exist in *A. orientalis*.

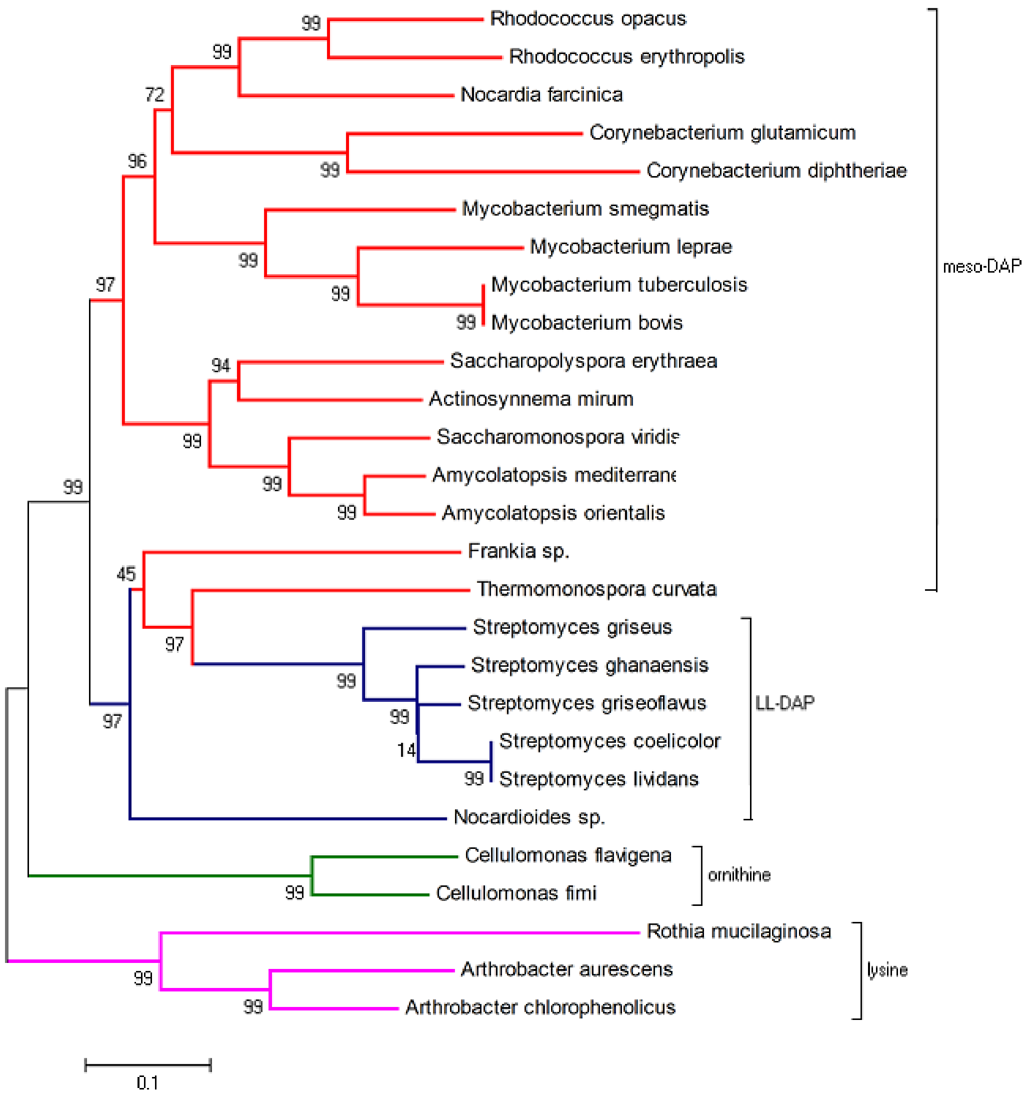

Figure S2. Pylogenetic analyses of MurE in actinomycetes. The amino acid sequences of the MurE are from 27 selected species color-coded based on the their special amino acid compositions of corresponding peptidoglycan: red, containing *meso*-diaminopimelic acid (DAP); blue, containing LL-DAP; green, containing lysine; pink, containing ornithine. The numbers on the internal branches are the percentage of bootstrap probability. The protein sequences were obtained from NCBI at http://www.ncbi.nlm.nih.gov/protein/. The phylogenetic relationship of the MurE protein from *A. orientalis* (AORI_2293) presents close relationship with those from *A. mediterranei* and *S. erythraea*, both of which synthesize cell wall using meso-DAP as the substrate. It is clearly distinguished from that of streptomycetes where LL-DAP is used as the substrate.

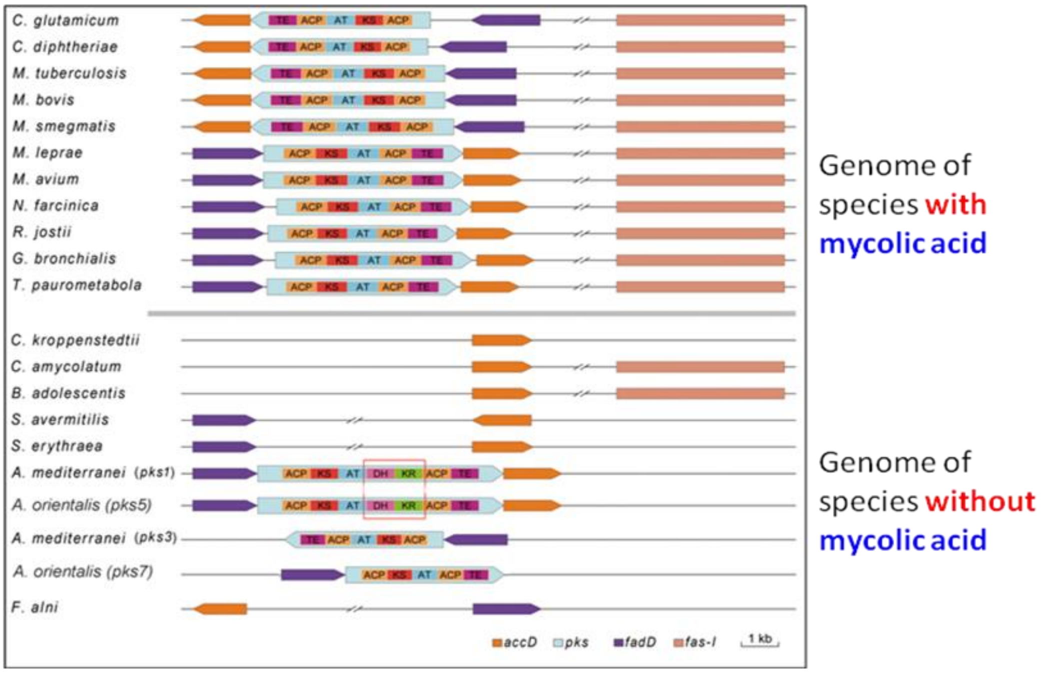

Figure S3. Genetic organization of the *fadD-pks-accD* and *fas-I* gene clusters in 20 selected actinobacterial genomes. The result was obtained by BLASTP using FadD32-PKS13-AccD4 and Fas-I of *M. tuberculosis* H37Rv as the query sequences. 11 strains above the gray bar are mycolic acids-containing bacteria that harbor both *fadD-pks-accD* clusters and *fas-I* genes. The remaining seven strains lack the mycolic acids in their cell envelope. The genomes used are as follow: *Corynebacterium glutamicum* ATCC 13032 (NC_003450), *Corynebacterium diphtheriae* NCTC 13129 (NC_002935), *M. tuberculosis* H37Rv (NC_000962), *Mycobacterium bovis* AF2122/97 (NC_002945), *Mycobacterium smegmatis* str. MC2 155 (NC_008596), *M. leprae* TN (NC_002677), *Mycobacterium avium* 104 (NC_008595), *Nocardia farcinica* IFM 10152 (NC_006361), *Rhodococcus jostii* RHA1 (NC_008268), *Gordonia bronchialis* DSM 43247 (NC_013441), *Tsukamurella paurometabola* DSM 20162 (NZ_ABVA00000000), *Corynebacterium kroppenstedtii* DSM 44385 (NC_012704), *Corynebacterium amycolatum* SK46 (NZ_ABZU00000000), *Bifidobacterium adolescentis* ATCC 15703 (NC_008618), *Streptomyces avermitilis* MA-4680 (NC_003155), *Saccharopolyspora erythraea* NRRL 2338 (NC_009142), *Amycolatopsis mediterrabei* U32 (NC_014318), *Frankia alni* ACN14a (NC_008278). This analysis indicates that, no authentic gene cluster of *fadD-pks-accD* critical for synthesizing mycolic acid component was found in the whole genome of *A. orientalis*, nor in the genome of *A. mediterranei*. It confirmed the characteristic phenotype of lacking mycolic acid in the cell wall of genus *Amycolatopsis*.

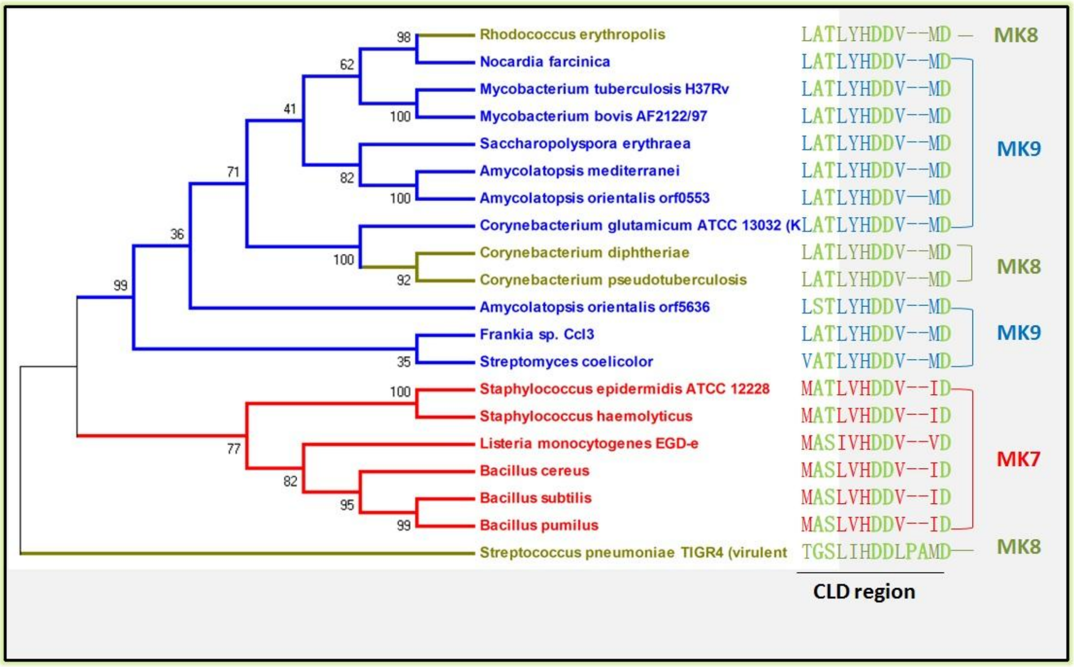

Figure S4. phylogenetic analysis of isoprenyl diphosphate synthases from type strains of actinomycetes using the MP method. The names and amino acid sequences of the strains with different colors represent actinomycetes harboring different-length MKs: red, MK7 (C35); olive-green, MK8 (C40); blue, MK9 (C45). The amino acid sequences of the chain-length determination (CLD) region are emphasized in green on the right of the panel. The protein sequences were obtained from NCBI at http://www.ncbi.nlm.nih.gov/protein/.

Table S5. The orthologs of key CDSs for secondary metabolite biosynthesis in *A.orientalis* HCCB10007 genome compared with the NCBI database.

A. orientalis HCCB10007					Best hits in the NCBI database			
Cluster	AORI_CDs	location	predicted domain	products/preliminary products	Orthologs	strain	protein	identity%
nrps1(vcm) (P1 in figure 3A)	AORI_1478	core	A-T-C-A-T-E-C-A-T	Leu-Tyr-Asn	lcl|Y16952.3_cdsid_CAC48360.1	*Amycolatopsis balhimycina* DSM5908	peptide synthetase	81
	AORI_1479		C-A-T-E-C-A-T-E-C-A-T	Hpg-Hpg-Tyr	lcl|Y16952.3_cdsid_CAC48361.1	*Amycolatopsis balhimycina* DSM5908	peptide synthetase	82
	AORI_1480		C-A-T-C-TE	Dpg	lcl|Y16952.3_cdsid_CAC48362.1	*Amycolatopsis balhimycina* DSM5908	peptide synthetase	87
car	AORI_1550	core		Carotene	AMED_4070 (*car*)	*Amycolatopsis mediterranei* U32	crtU	83
	AORI_1551				AMED_4071	*Amycolatopsis mediterranei* U32	ubiE	80
	AORI_1552				AMED_4072	*Amycolatopsis mediterranei* U32	lycopene cyclase	77
	AORI_1553				AMED_4073	*Amycolatopsis mediterranei* U32	lycopene cyclase	81
	AORI_1554				AMED_4074	*Amycolatopsis mediterranei* U32	crtB	80
	AORI_1555				AMED_4075	*Amycolatopsis mediterranei* U32	crtI	85
	AORI_1556				AMED_4076	*Amycolatopsis mediterranei* U32	idsA	79
pks1(pksE)	AORI_1833	core			AMED_6872 (*pks4*)	*Amycolatopsis mediterranei* U32	enediyne biosynthesis protein	79
	AORI_1840		KS-AT-KR		AMED_6864	*Amycolatopsis mediterranei* U32	PKS	80
	AORI_1843				AMED_6859	*Amycolatopsis mediterranei* U32	propionyl-CoA carboxylase	48
lyc	AORI_2309	core		Lycopene	AMED_2322 (*lyc*)	*Amycolatopsis mediterranei* U32	crtB	74
	AORI_2310				AMED_2323	*Amycolatopsis mediterranei* U32	crtI	80
	AORI_2311				AMED_2324	*Amycolatopsis mediterranei* U32	idsA	87
nrps2	AORI_2921	non core	C-A-T-C-T-TE-MFS	?	gi|271965573|ref|YP_003339769.1|	*Streptosporangium roseum* DSM 43021	non-ribosomal peptide synthetase-like protein	50
	AORI_2922		A-T-C-A-T-C-A-T	Arg-?-?	gi|158339498|ref|YP_001520887.1|	*Acaryochloris marina* MBIC11017	peptide synthetase, putative	35
pks2(eco)	AORI_2945	non core	ACP-KS-AT-DH-KR-ACP -KS-AT-DH-ER-KR-ACP		gi|216409659|dbj|BAH02268.1|	*Streptomyces platensis*	polyketide synthase type I	56
	AORI_2946		KS-AT-DH-KR-ACP-KS-AT-DH-ER-KR-ACP-KS-AT-DH-ER-KR-ACP		gi|345015244|ref|YP_004817598.1|	*Streptomyces violaceusniger* Tu 4113	polyketide synthase type I	57
	AORI_2947		KS-AT-DH-KR-ACP		gi|15824140|dbj|BAB69304.1|	*Streptomyces avermitilis*	polyketide synthase type I	55
	AORI_2948		KS-AT-KR-ACP-KS-AT-DH-KR-ACP		gi|345011738|ref|YP_004814092.1|	*Streptomyces violaceusniger* Tu 4113	polyketide synthase type I	55
	AORI_2949		KS-AT-DH-KR-ACP-KS-AT-DH-KR-ACP-KS-AT-DH-KR-ACP		gi|323435190|gb|ADX66472.1|	*Streptomyces chattanoogensis*	polyketide synthase type I	57
	AORI_2950		KS-AT-DH-KR-ACP-TE		gi|306407929|dbj|BAJ16471.1|	*Streptomyces graminofaciens*	polyketide synthase type I	53
n_p1	AORI_3262	non core	A-T-KS	Phe	gi|302867256|ref|YP_003835893.1|	*Micromonospora aurantiaca* ATCC 27029	amino acid adenylation domain-containing protein	65
	AORI_3263		A-T-C-T	Trp	gi|302867259|ref|YP_003835896.1|	*Micromonospora aurantiaca* ATCC 27029	amino acid adenylation domain-containing protein	60
	AORI_3264		KS-AT-KR-ACP		gi|271963948|ref|YP_003338144.1|	*Streptosporangium roseum* DSM 43021	polyketide/non-ribosomal peptide synthetase	64
	AORI_3265		C-A-T	Asn	gi|271963946|ref|YP_003338142.1|	*Streptosporangium roseum* DSM 43021	non-ribosomal peptide synthetase/polyketide synthase	58
	AORI_3266		C-A-T-TE-MFS	Ser	gi|271963945|ref|YP_003338141.1|	*Streptosporangium roseum* DSM 43021	peptide synthetase	68
pks3	AORI_3405	non core			AMED_3367 (*pks1*)	*Amycolatopsis mediterranei* U32	FadD	
	AORI_3406		ACP-KS-AT-DH-KR-ACP -TE		AMED_3368	*Amycolatopsis mediterranei* U32	typeI polyketide synthase	67
nrps3	AORI_3539	non core	A	?	gi|257056094|ref|YP_003133926.1|	*Saccharomonospora viridis* DSM 43017	peptide arylation enzyme	81
	AORI_3547		T		gi|345003770|ref|YP_004806624.1|	*Streptomyces sp.* SirexAA-E	amino acid adenylation domain-containing protein	67
nrps4	AORI_3675	quasi core	C-A-T-C-A-T-C-A-M-T-C-A-T-C-A-T-C-A-T-E	Ser-Gln-Ser-Thr-Ser-Ala	gi|271964951|ref|YP_003339147.1|	*Streptosporangium roseum* DSM 43021	non-ribosomal peptide synthetase-like protein	61
nrps5	AORI_4035	non core	A-T	?	gi|119952361|ref|YP_950027.1|	*Arthrobacter aurescens* TC1	putative non-ribosomal peptide synthetase	56

tps1	AORI_4062	non core		Terpene	AMED_3240 (*tps2*)	*Amycolatopsis mediterranei* U32	terpene synthase	67
nrps6	AORI_4540	non core	C-A-T-C-T-C-A-T	Arg-Ahp	gi|159900486|ref|YP_001546733.1|	*Herpetosiphon aurantiacus* DSM 785	amino acid adenylation domain-containin g protein	35
	AORI_4541		C-A-T-C-A-T-TE	Thr	gi|256377653|ref|YP_003101313.1|	*Actinosynnema mirum DSM* 43827	amino acid adenylation domain-containin g protein	39
pks4	AORI_4645	non core	KS-AT-DH-ER-KR-ACP-T E		gi|297153560|gb|ADI03272.1|	*Streptomyces bingchenggensis* BCW-1	putative type-I PKS	44
nrps7	AORI_4675	non core			AMED_3103 (*nrps1*)	*Amycolatopsis mediterranei* U32	MbtH	57
	AORI_4676				AMED_3106	*Amycolatopsis mediterranei* U32	MFT	71
	AORI_4677		C-A-T-C-A-T-E-C-A-T-C-A-T-E	Trp-Thr-Thr-?	AMED_3105	*Amycolatopsis mediterranei* U32	NRPS	53
	AORI_4678		A-T-C-A-T-E-C-A-T-E-C-A-T-E-C-A-T	Val-Val-Val -Val-Val	AMED_3102	*Amycolatopsis mediterranei* U32	NRPS	45
pks5	AORI_4816	non core	TE		AMED_4485 (*pks2*)	*Amycolatopsis mediterranei* U32	esterase	79
	AORI_4818				AMED_4483	*Amycolatopsis mediterranei* U32	acyl-CoA synthetase	77
	AORI_4819		ACP-KS-AT-ACP -TE		AMED_4482	*Amycolatopsis mediterranei* U32	PKS	68
pks6	AORI_4851	non core			AMED_4606 (*pks3*)	*Amycolatopsis mediterranei* U32	ACT	75
	AORI_4852		KS-AT		AMED_4605	*Amycolatopsis mediterranei* U32	PKS	80
	AORI_4853		KS-AT-KR		AMED_4604	*Amycolatopsis mediterranei* U32	PKS	83
nrps8	AORI_5017	non core	A-T-Bacterial transferase hexapeptide (six repeats)	?	gi|302527997|ref|ZP_07280339.1|	*Streptomyces sp.* AA4	non-ribosomal peptide synthetase	87
n_p2	AORI_5325	non core	A-T-TE	?	gi|134099557|ref|YP_001105218.1|	*Saccharopolyspora erythraea* NRRL 2338	non-ribosomal peptide synthetase	45
	AORI_5330		Chalcone synthases		gi|328880541|emb|CCA53780.1|	*Streptomyces venezuelae* ATCC 10712	Chalcone synthase	66
	AORI_5331		C		gi|108762076|ref|YP_631821.1|	*Myxococcus xanthus* DK 1622	non-ribosomal peptide synthase	39
	AORI_5337		C		gi|354565110|ref|ZP_08984286.1|	*Fischerella sp.* JSC-11	amino acid adenylation domain protein	34
	AORI_5340		A-T-Cy-A-T	?	gi|284032053|ref|YP_003381984.1|	*Kribbella flavida* DSM 17836	amino acid adenylation domain-containin g protein	53
	AORI_5344		A-T	Me-Pro	gi|284032033|ref|YP_003381964.1|	*Kribbella flavida* DSM 17836	amino acid adenylation domain-containin g protein	44
	AORI_5345		TE		gi|284032033|ref|YP_003381964.1|	*Kribbella flavida* DSM 17836	amino acid adenylation domain-containin g protein	42
	AORI_5346		C-T		gi|284032032|ref|YP_003381963.1|	*Kribbella flavida* DSM 17836	N-(5-amino-5-car boxypentanoyl)-L -cysteinyl-D-valin e synthase	44
	AORI_5347		C-A-T	Phe	gi|284032030|ref|YP_003381961.1|	*Kribbella flavida* DSM 17836	amino acid adenylation domain-containin g protein	39
	AORI_5348		TE		gi|302547201|ref|ZP_07299543.1|	*Streptomyces hygroscopicus* ATCC 53653	pyochelin biosynthetic protein PchC	45
	AORI_5349		A-T	Thr	gi|297197053|ref|ZP_06914450.1|	*Streptomyces sviceus* ATCC 29083	non-ribosomal peptide synthetase	51
nrps9	AORI_5362	non core	TE		gi|330468934|ref|YP_004406677.1|	*Verrucosispora maris* AB-18-032	thioesterase	37
	AORI_5363		A-T	Gln	gi|556565397|ref|YP_008733822.1|	*Actinoplanes friuliensis*	non-ribosomal peptide synthetase	45
pks7	AORI_5390	non core			gi|11595497|emb|CAC18323.1|	*Amycolatopsis sp.* HR167	feruloyl-CoA synthetase	60
	AORI_5391		A-T-KS-AT-DH-KR-ACP-TE		gi|297156588|gb|ADI06300.1|	*Streptomyces bingchenggensis* BCW-1	type I modular polyketide synthase	67
	AORI_5395				gi|21225119|ref|NP_630898.1|	*Streptomyces coelicolor* A3(2)	polyketide synthase	48
	AORI_5397		KS-AT-DH-KR-ACP		gi|224812393|gb|ACN64831.1|	*Streptomyces diastatochromogenes*	PokM1	48
	AORI_5398				gi|217978970|ref|YP_002363117.1|	*Methylocella silvestris* BL2	putative acyl-CoA dehydrogenase	43
pks8	AORI_5439	non core	short chain dehydrogenase-KS -KS*-KR		gi|311893816|dbj|BAJ26224.1|	*Kitasatospora setae* KM-6054	putative fatty acid synthase	41
	AORI_5440		AT-ACP		gi|311893815|dbj|BAJ26223.1|	*Kitasatospora setae* KM-6054	beta-ketoacyl synthase	42
n_p3	AORI_5501	non core	short chain dehydrogenase-KS -KS*		gi|167840976|ref|ZP_02467660.1|	*Burkholderia thailandensis* MSMB43	polyketide synthase, putative	43
	AORI_5502		KR-DH-KR		gi|167840976|ref|ZP_02467660.1|	*Burkholderia thailandensis* MSMB43	polyketide synthase, putative	34
	AORI_5505		ACP		gi|291435672|ref|ZP_06575062.1|	*Streptomyces ghanaensis* ATCC 14672	acyl carrier protein	45
	AORI_5506		A	?	gi|45580865|emb|CAG15025.1|	*Actinoplanes teichomyceticus*	adenylation protein	45
nrps10	AORI_6000	core	A-T-TE	?	gi|302545361|ref|ZP_07297703.1|	*Streptomyces hygroscopicus* ATCC 53653	non-ribosomal peptide synthetase	61

	AORI_6001		TE		gi|337769426|emb|CCB78139.1|	*Streptomyces cattleya* NRRL 8057	esterase	49
pks9 (P4 in figure 3A)	AORI_6604	core	KS*		gi|330465416|ref|YP_004403159.1|	*Verrucosispora maris* AB-18-032	beta-ketoacyl synthase	53
	AORI_6605		KS		gi|145595315|ref|YP_001159612.1|	*Salinispora tropica* CNB-440	beta-ketoacyl synthase	60
	AORI_6606		KS*		gi|145595316|ref|YP_001159613.1|	*Salinispora tropica* CNB-440	beta-ketoacyl synthase	56
	AORI_6607		KS		gi|145595317|ref|YP_001159614.1|	*Salinispora tropica* CNB-440	beta-ketoacyl synthase	74
	AORI_6608		ACP		gi|330465412|ref|YP_004403155.1|	*Verrucosispora maris* AB-18-032	phosphopantethei ne-binding protein	63
	AORI_6609				gi|145595319|ref|YP_001159616.1|	*Salinispora tropica* CNB-440	beta-hydroxyacyl-(acyl-carrier-prote in) dehydratase, FabA/FabZ	67
	AORI_6610				gi|330465410|ref|YP_004403153.1|	*Verrucosispora maris* AB-18-032	3-hydroxyacyl-A CP dehydratase	52
	AORI_6611				gi|145595321|ref|YP_001159618.1|	*Salinispora tropica* CNB-440	3-oxoacyl-(acyl-c arrier-protein) reductase	73
	AORI_6613				gi|330465407|ref|YP_004403150.1|	*Verrucosispora maris* AB-18-032	acetyl-CoA acetyltransferase	74
	AORI_6614				gi|330465392|ref|YP_004403135.1|	*Verrucosispora maris* AB-18-032	acyl-CoA dehydrogenase domain-containin g protein	69
	AORI_6615				gi|330465391|ref|YP_004403134.1|	*Verrucosispora maris* AB-18-032	acyl-CoA dehydrogenase domain-containin g protein	53
tps2	AORI_7417	core		Terpene	AMED_1325 (*tps1*)	*Amycolatopsis mediterranei* U32	terpene synthase	55

Note: All of the polyketide synthases, the nonribosomal peptide-synthetases, or the terpene synthases in total 26 biosynthetic gene clusters were employed to run the BLASTP program comparing against to the NCBI database and the best hits information was listed. The domain organization of PKS/NRPS enzymes and the putative precursors of NRPSs are analyzed on the SBSPKS or NRPSDB database. Nine clusters of *car*, *pks1*, *lyc*, *pks3*, *tps1*, *nrps7*, *pks5*, *pks6* and *tps2* have their orthologs in the *A. mediterranei* genome with the highest hitting scores, respectively.

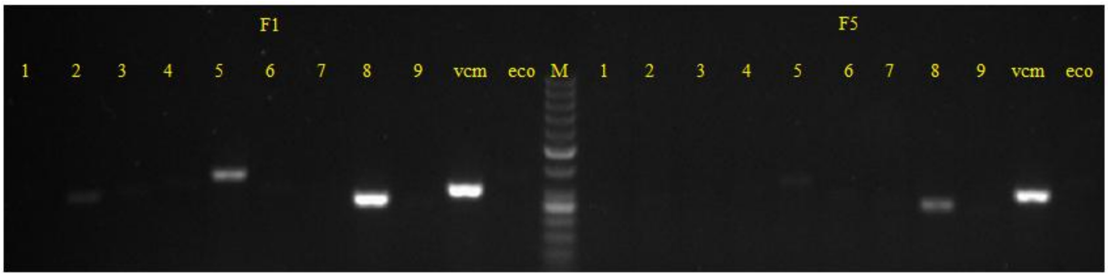

Figure S5. The reverse-transcription PCR of RNA isolated from different cultures (poor medium F1 and rich medium F5). Line 1 to 9 represents the genes of *nrps3*, *nrps4*, *nrps7*, *n_p1*, *n_p2*, *n_p3*, *pks3*, *pks5*, *pks9*, respectively. Line *vcm* and *eco* represent the genes of vancomycin biosynthetic cluster and *eco* polyketide cluster.

Table S6. Genes encoded for racemases in *A.orientalis* HCCB10007 genome

AORI_CDS	Strand	Racemase_name	Location
**AORI_0075**	+	amino acid aldolase or racemase	core region
**AORI_0725**	+	alanine racemase	core region
**AORI_2199**	-	fatty acid-CoA racemase	core region
**AORI_2442**	+	alpha-methylacyl-CoA racemase	core region
**AORI_3621**	+	proline racemase	non-core region
**AORI_4003**	+	mandelate racemase/muconate lactonizing enzyme-like protein	non-core region
**AORI_5039**	+	Asp/Glu/hydantoin racemase	quasi-core region
**AORI_6044**	-	mandelate racemase	core region
**AORI_6304**	+	enoyl-CoA hydratase/carnithine racemase	core region
**AORI_6488**	-	glutamate racemase	core region
**AORI_6491**	-	aspartate racemase	core region

Table S7. NMR spectroscopic data for dimethylvancomycin (see the chemical structural formula below)

	dimethylvancomycin[a]			vancomycin[b]		
NO.	δ_H	δ_C	NO.	δ_H	δ_C	group
1	1.18 (d, 6.3)	18.4	1	/	/	CH_3
2	0.96 (d, 6.3)	22.7	2	/	/	CH_3
3	0.92 (d, 6.1)	23.2	3	/	/	CH_3
4	1.17 (s)	24.5	4	/	/	CH_3
5	1.28 (m 7.4)	26.8	5	/	/	CH
6	2.14 (br m)<br>2.38(br d, 7.3)	35.4	6	/	/	CH_2
7	1.83 (br d,14.8, 5.6)<br>2,29 (br m)	37.9	7	/	/	CH_2
8	1.52 (m)<br>1.69 (m)	38.8	8	/	/	CH_2
8’	2.91 (s)	43.9	8’	2.37(s)	33.2	CH_3
**8’’**	**2.91(s)**	**43.9**	**c**	–	–	$\mathbf{CH_3}$
9	3.2 (br m)	49.3	9	/	/	CH
10	3.82 (m)	63.3	10	/	/	CH
11	4.78 (s)	54.0	11	/	/	CH
12		56.5	12	/	/	C
13	4.56 (s)	57.1	13	/	/	CH
14	5.84 (s)	57.6	14	/	/	CH
15	5.58 (m)	61.0	15	/	/	CH
16	5.40 (dd, 16.3, 7.6)<br>5.58(m)	62.1	16	/	/	CH
17	5.56(m)	62.9	17	/	/	CH
18	3.65 (m)	71.9	18	/	/	CH
19	4.83 (br d, 1.0)	73.2	19	/	/	CH
20	4.92(br d, 11.5)	73.8	20	/	/	CH
21	4.85(o)	74.0	21	/	/	CH
22	4.82 (o)	74.1	22	/	/	CH
23	5.40 (m)	78.5	23	/	/	CH
24	5.38 (m)	79.0	24	/	/	CH
25	4.82 (o)	81.6	25	/	/	CH
26	4.85 (o)	100.0	26	/	/	CH
27	5.41 (m)	103.8	27	/	/	CH
28	6.32 (d, 2.1)	105.4	28	/	/	CH
29	5.25 (d, 1.4)	108.3	29	/	/	CH
30	6.58 (d, 2.1)	107.0	30	/	/	CH
31	5.45 (m)	108.3	31	/	/	CH
32	6.70 (d, 8.2)	120.1	32	/	/	CH

33		120.6	33	/	/	C
34		123.8	34	/	/	C
35	7.35 (d, 8.1)	125.2	35	/	/	CH
36	7.24 (d, 8.2)	126.2	36	/	/	CH
37	6.58 (d, 8.1)	126.8	37	/	/	CH
38		127.8	38	/	/	C
39		128.6	39	/	/	C
40		128.7	40	/	/	C
41	7.73 (d, 8.2)	129.0	41	/	/	CH
42	7.71 (d, 8.2)	129.7	42	/	/	CH
43	7.76 (s)	130.2	43	/	/	CH
44	7.61 (s)	130.6	44	/	/	CH
45		131.2	45	/	/	C
46		131.6	46	/	/	C
47	7.47 (br s)	135.0	47	/	/	CH
48		138.0	48	/	/	C
49		140.7	49	/	/	C
50		141.1	50	/	/	C
51		142.5	51	/	/	C
52		142.9	52	/	/	C
53		144.5	53	/	/	C
54		151.5	54	/	/	C
55		153.1	55	/	/	C
56		155.2	56	/	/	C
57		156.7	57	/	/	C
58		157.3	58	/	/	C
59		158.9	59	/	/	C
60		170.4	60	/	/	C
61		170.5	61	/	/	C
62		173.0	62	/	/	C
63		173.5	63	/	/	C
64		176.6	64	/	/	C
65		179.1	65	/	/	C

a: ^{1}H-NMR was measured at 500 MHz in D_2O, and ^{13}C-NMR was measured at 125 MHz in D_2O.

b: The NMR data for vancomycin was studied by Pearce (1995).

c: Compared with that of vancomycin, the NMR data for dimethylvancomycin shows another methyl-group at the position of 8’’ was added.

-: absent; /: identical

Table S8. NMR spectroscopic data for dimethylaglucovancomycin (see the chemical structural formula below)

NO.	dimethylaglucovancomycin[a]		NO.	vancomycin[b]		group
	δ_H	δ_C		δ_H	δ_C	
1	0.87 (d, 3.0)	22.7	1	/	/	CH_3
2	0.86 (d, 2.96)	23.8	2	/	/	CH_3
3	1.64(m)	25.4	3	/	/	CH
4	1.27(m) 1.55(m)	35.8	4	/	/	CH_2
5	2.12(dd,16.24, 5.05) 2.29(m)	39.3	5	/	/	CH_2
5’	2.29(s)	42.2	5’	2.37(s)	33.2	CH_3
**5’’**	**2.29(s)**	**42.2**	**c**	**-**	**-**	**CH_3**
6	4.49(m)	51.3	6	/	/	CH
7	4.43 (d, 5.68)	55.0	7	/	/	CH
8	4.45(s)	57.2	8	/	/	CH
9	4.18 (d, 11.96)	62.3	9	/	/	CH
10	4.88(q)	58.7	10	/	/	CH
11	3.03(m)	67.1	11	/	/	CH
12	5.71(s)	54.2	12	/	/	CH
13	5.19 (d, 4.32)	71.3	13	/	/	CH
14	5.10 (d, 5.56)	72.1	14	/	/	CH
15	6.40 (d, 1.92)	102.8	15	/	/	CH
16	5.20 (d, 4.32)	104.9	16	/	/	CH
17	6.27(s)	106.2	17	/	/	CH
18	5.50(s)	107.3	18	/	/	CH
19	6.71 (d, 8.6)	116.7	19	/	/	CH
20		118.5	20	/	/	C
21		122.1	21	/	/	C
22	7.22 (d, 15.36)	123.8	22	/	/	CH
23	7.33 (d, 8.36)	125.0	23	/	/	CH
24	6.78 (d, 8.6)	126.0	24	/	/	CH
25		126.6	25	/	/	C
26		126.8	26	/	/	C
27		127.5	27	/	/	C
28	7.46 (dd, 15.36,8.36)	127.6	28	/	/	CH
29	7.50 (dd, 15.36, 8.36)	127.8	29	/	/	CH
30	7.83(s)	128.0	30	/	/	CH
31	7.28(m)	128.7	31	/	/	CH
32		129.3	32	/	/	C
33		134.4	33	/	/	C
34	7.18(s)	136.1	34	/	/	CH
35		136.7	35	/	/	C

36	140.0	36	/	/	C
37	142.6	37	/	/	C
38	147.8	38	/	/	C
39	148.0	39	/	/	C
40	149.4	40	/	/	C
41	151.0	41	/	/	C
42	155.5	42	/	/	C
43	157.0	43	/	/	C
44	157.6	44	/	/	C
45	168.1	45	/	/	C
46	168.3	46	/	/	C
47	169.7	47	/	/	C
48	170.8	48	/	/	C
49	171.0	49	/	/	C
50	171.1	50	/	/	C
51	172.5	51	/	/	C
52	173.0	52	/	/	C

a: ^{1}H-NMR was measured at 400 MHz in DMSO-d_6, and ^{13}C-NMR was measured at 100 MHz in DMSO-d_6.

b: The NMR data for vancomycin was studied by Pearce (1995).

c: Compared with that of vancomycin, the NMR data for dimethylaglucovancomycin shows another methyl-group at the position of 5’’ was added. Differences include the lack of a glucosyl- group and a vancosamine- group in dimethylaglucovancomycin (data not shown).

-: absent; /: identical

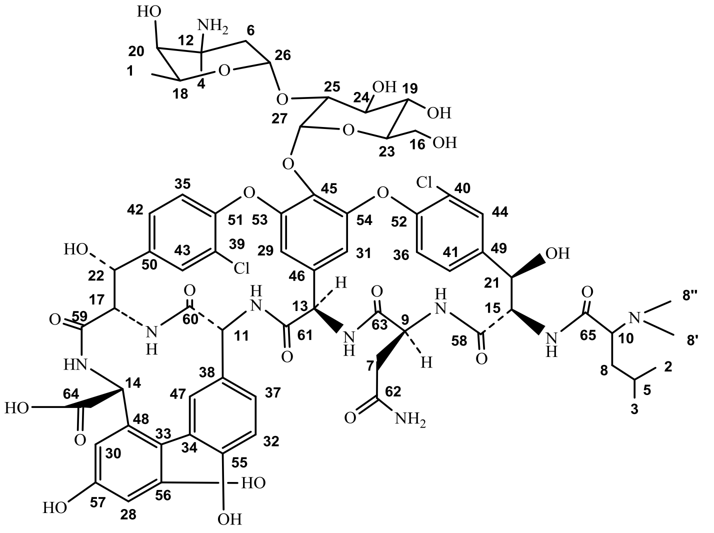

dimethylvancomycin

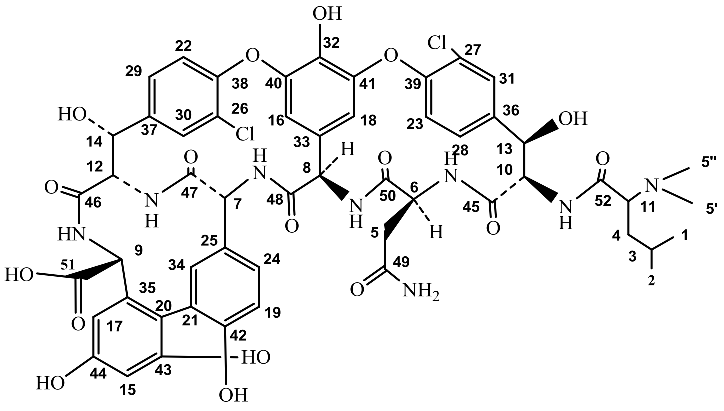

dimethylaglucovancomycin
